# The nephroprotective action of *Passiflora edulis* in streptozotocin-induced diabetes

**DOI:** 10.1038/s41598-022-21826-9

**Published:** 2022-10-20

**Authors:** Ony Araújo Galdino, Iago de Souza Gomes, Renato Ferreira de Almeida Júnior, Maria Imaculada Conceição Ferreira de Carvalho, Bento João Abreu, Marcela Abbott Galvão Ururahy, Barbara Cabral, Silvana Maria Zucolotto Langassner, Karla Simone Costa de Souza, Adriana Augusto de Rezende

**Affiliations:** 1 Department of Clinical and Toxicological Analyses, Federal University of Rio Grande do Norte, Av. General Gustavo Cordeiro de Farias, S/N, Faculty of Pharmacy, Petrópolis, Natal, RN CEP: 59012-570 Brazil; 2 Department of Morphology, Federal University of Rio Grande do Norte, Natal, RN Brazil; 3 Department of Pharmacy, Federal University of Rio Grande do Norte, Natal, RN Brazil

**Keywords:** Molecular biology, Diabetes

## Abstract

In the present study, we aimed to evaluate the therapeutic effect of *Passiflora edulis* fruit peel aqueous (AFA) extract as an adjuvant to insulin to confer nephroprotection against streptozotocin-induced diabetes. Male Wistar rats were divided into four groups based on treatment received for 60 days: diabetic (DB), control (CTL), insulin (INS), and insulin + AFA extract (INS + AFA). mRNA and protein expression levels of podocyte (nephrin, podocin, and WT1) and tubular (megalin) proteins were measured in kidney tissue specimens and urine. Biochemical parameters and kidney histopathology were also examined. Herein, the INS + AFA group showed superior glycemic control, which resulted in the reduction of urinary albumin/creatinine ratio, maintenance of baseline levels of *Nphs1, Nphs2, Wt1,* and *Lrp2* mRNA expression, prevention of protein loss from the kidney tissue into the urinary space, along with the maintenance of glomerular basement membrane thickness, hyalinization, glomerular and tubulointerstitial fibrosis at values approximating those of the CTL group and significantly lower than those in the DB group. Therefore, these results suggest that, as an anti-diabetic agent, the AFA extract adjuvant to insulin could reduce and potentially prevent diabetic kidney disease.

## Introduction

Diabetic kidney disease (DKD) is a frequent and severe microvascular complication resulting from damage to glomeruli and renal tubules. DKD is mediated by morphological changes in kidney tissue, including thickening of the glomerular basement membrane (GBM), mesangial cell expansion, extracellular matrix deposition, and podocyte damage or loss^1,2^. These changes trigger disturbances in essential kidney function, such as electrolyte imbalance, decreased glomerular filtration rate (GFR), increased blood pressure, and elevated urinary protein excretion^3^.

DKD-induced morphological changes lead to the loss of podocytes and tubular proteins in the urinary space. Proteins that comprise the kidney tissue, such as nephrin, podocin, Wilms' tumor-1 (WT1), and megalin, can indicate early kidney damage when present in the urine prior to the appearance of albuminuria^4,5^. Thus, the presence of these proteins in the urine makes them particularly useful for assessing early kidney damage^6–9^.

Regarding therapeutic interventions, insulin can delay the onset and progression of DKD by affording glycemic control; however, in some cases, insulin monotherapy is insufficient to maintain blood glucose within the therapeutic target, requiring adjuvant therapeutic interventions^10–12^. Considering that available drugs can induce several adverse effects in patients, studies have attempted to identify alternative drugs from natural sources capable of controlling glycemic levels and reducing complications such as DKD^13^.

Among the various species that can be valuable for treating diabetes, we focused on Passiflora *edulis* (*P. edulis*) f. *flavicarpa* O. Deg. (*Passifloraceae*), popularly known as the yellow passion fruit. Previous pharmacological studies have reported the *P. edulis*-mediated anti-diabetic, anti-hypertensive, and anti-inflammatory agent effects, in addition to other effects such as the attenuation of diabetes complications^14–17^.

Herein, we aimed to evaluate the therapeutic effect of *P. edulis* fruit peel aqueous (AFA) extract adjuvant to insulin as a potential nephroprotective agent against streptozotocin-induced diabetes.

## Results

### Biochemical analyses and body weight

Table 1 presents the body weight and results of biochemical analysis. The baseline body weight at study initiation was similar across all groups (average 220–250 g). However, after 60 days of experimentation, the body weight was maintained in the control (CTL), insulin (INS), and insulin + AFA extract (INS + AFA) groups when compared with the diabetic (DB) group (P < 0.001 for all).

**Table 1:** Body weight and biochemical analyses of control, diabetic, and treated diabetic groups.

Variables	Control	Diabetic	Insulin	Insulin + aqueous extract (AFA)
Body weight, g	300 (283–369)	165 (153–169)^#^	306 (285–332)*	263 (250–275)^#^*^§^
Glucose, mg/dL	127 (97–138)	546 (470–688)^#^	89 (54–107)^#^*	50 (29–56)^#^*^§^
Serum creatinine, mg/dL	0.38 (0.34–0.42)	0.62 (0.44–0.89)^#^	0.50 (0.40–0.65)^#^	0.37 (0.33–0.39)*^§^
ACR, mg/g of creatinine	22.50 (20.30–26.20)	724.30 (583.80–1490.80)^#^	105.70 (78.30–248.80)^#^*	53.60 (42.50–187)^#^*
NGAL, ng/mL	0.35 (0.32–0.40)	0.25 (0.14–7.51)	0.20 (0.15–0.30)	0.15 (0.13–0.19)
Total serum proteins, g/dL	6.11 (5.98–6.27)	5.18 (4.88–5.49)^#^	6.03 (5.87–6.10)*	5.95 (5.76–5.97)*
Serum albumin, g/dL	2.59 (2.56–2.66)	1.97 (1.81–2.17)^#^	2.58 (2.51–2.62)*	2.68 (2.63–2.70)*

Results are expressed as the median (interquartile range).

*ACR* urinary albumin/creatinine ratio, *NGAL* neutrophil gelatinase-associated lipocalin.

^#^P < 0.05, vs. control group; *P < 0.05, diabetic group; ^§^P < 0.05, insulin group.

As expected, the serum glucose concentrations in the DB group were higher than those in the CTL group (P = 0.034). Compared with the DB group, we noted a significant reduction in blood glucose levels in the INS and INS + AFA groups (P = 0.027 and P < 0.001, respectively). Furthermore, a significant tenfold reduction in serum glucose concentration was documented in the INS + AFA group when compared with that in the INS group (P = 0.010).

Regarding the renal function parameters, the serum creatinine level was significantly higher in the DB group than in the CTL group (P = 0.034). A significant reduction in serum creatinine level was also observed in the INS + AFA group when compared with that in the INS group (P = 0.003). The urinary albumin/creatinine ratio (ACR) was significantly higher in the DB, INS, and INS + AFA groups than in the CTL group (P < 0.001, P = 0.034, and P < 0.001, respectively). Compared with the DB group, we noted a significant reduction in ACR in the INS (sevenfold) and INS + AFA (14-fold) groups (P = 0.036 and P = 0.005, respectively). Furthermore, ACR positively correlated with serum glucose levels (r = 0.622, P < 0.001). Urinary concentrations of neutrophil gelatinase-associated lipocalin (NGAL) did not differ significantly between examined groups.

Regarding serum proteins, the DB group showed a reduction in serum total protein and albumin concentrations when compared with the CTL group (P = 0.034 and P = 0.003, respectively). In addition, increased serum concentrations of total protein and albumin were detected in the INS (P = 0.027 and P = 0.001, respectively) and INS + AFA (P = 0.034 and P < 0.001, respectively) groups when compared with the DB group.

### Evaluation of mRNA expression in kidney tissue

**Figure 1:**
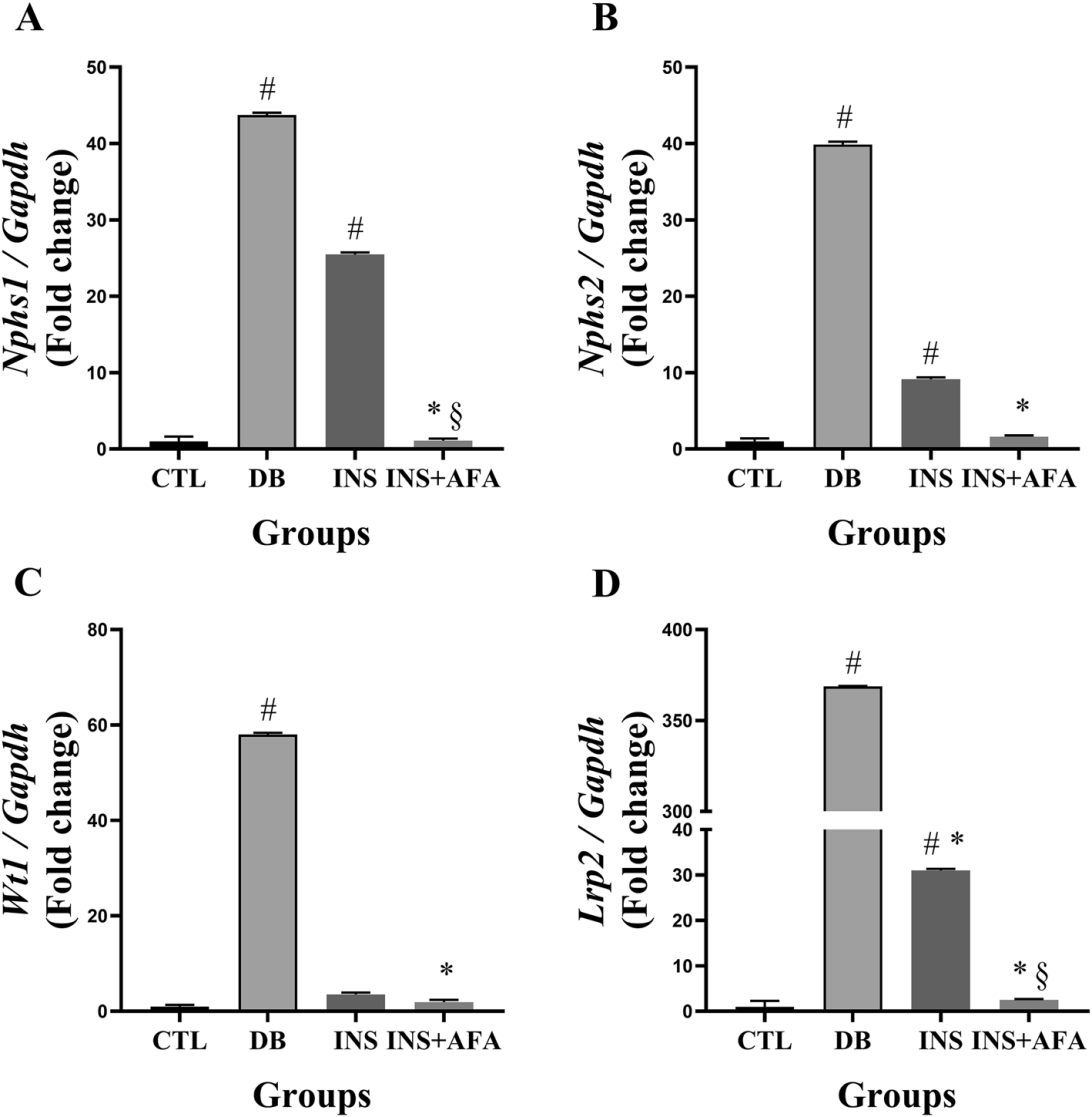
Quantification of relative mRNA expression in kidney tissues. *Nphs1* (**A**)*, Nphs2* (**B**)*, WT1* (**C**)*,* and *Lrp2* (**D**) mRNA expression in kidney tissues of the control, diabetic, and treated diabetic groups; data are expressed as fold-change vs. control group values, normalized to *Gapdh*; CTL, control (n = 11); DB, diabetic (n = 6); INS, insulin (n = 11); INS + AFA, insulin + aqueous extract of *P. edulis* (n = 9); ^#^P < 0.05 vs. control group; *P < 0.05 vs. diabetic group; ^§^P < 0.05 vs. insulin group.

Figure 1 presents mRNA expression data for the kidney tissue. Compared with the CTL group, the DB group showed increased mRNA expression of *Nphs1, Nphs2, Wt1*, and *Lrp2* (P = 0.001, for all). In addition, the mRNA expression of *Nphs1, Nphs2,* and *Lrp2* was higher in the INS group than in the CTL group (P = 0.001, P = 0.010, and P = 0.012, respectively).

However, the mRNA expression levels of *Nphs1, Nphs2, Wt1,* and *Lrp2* were reduced in the INS + AFA group when compared with the DB group (P = 0.004, P = 0.010, P = 0.006, and P = 0.004, respectively). Furthermore, the mRNA expression levels of *Nphs1* and *Lrp2* were lower in the INS + AFA group than in the INS group (P = 0.003 and P = 0.010, respectively). Interestingly, the mRNA expression levels of *Nphs1, Nphs2, Wt1,* and *Lrp2* in the INS + AFA group were similar to those in the CTL group.

Expression levels of *Nphs1* (r = 0.451, P = 0.009), *Nphs2* (r = 0.435, P = 0.015), and *Wt1* (r = 0.660, P < 0.001) showed significant positive correlations with serum glucose levels.

### Evaluation of protein expression in kidney tissue

**Figure 2:**
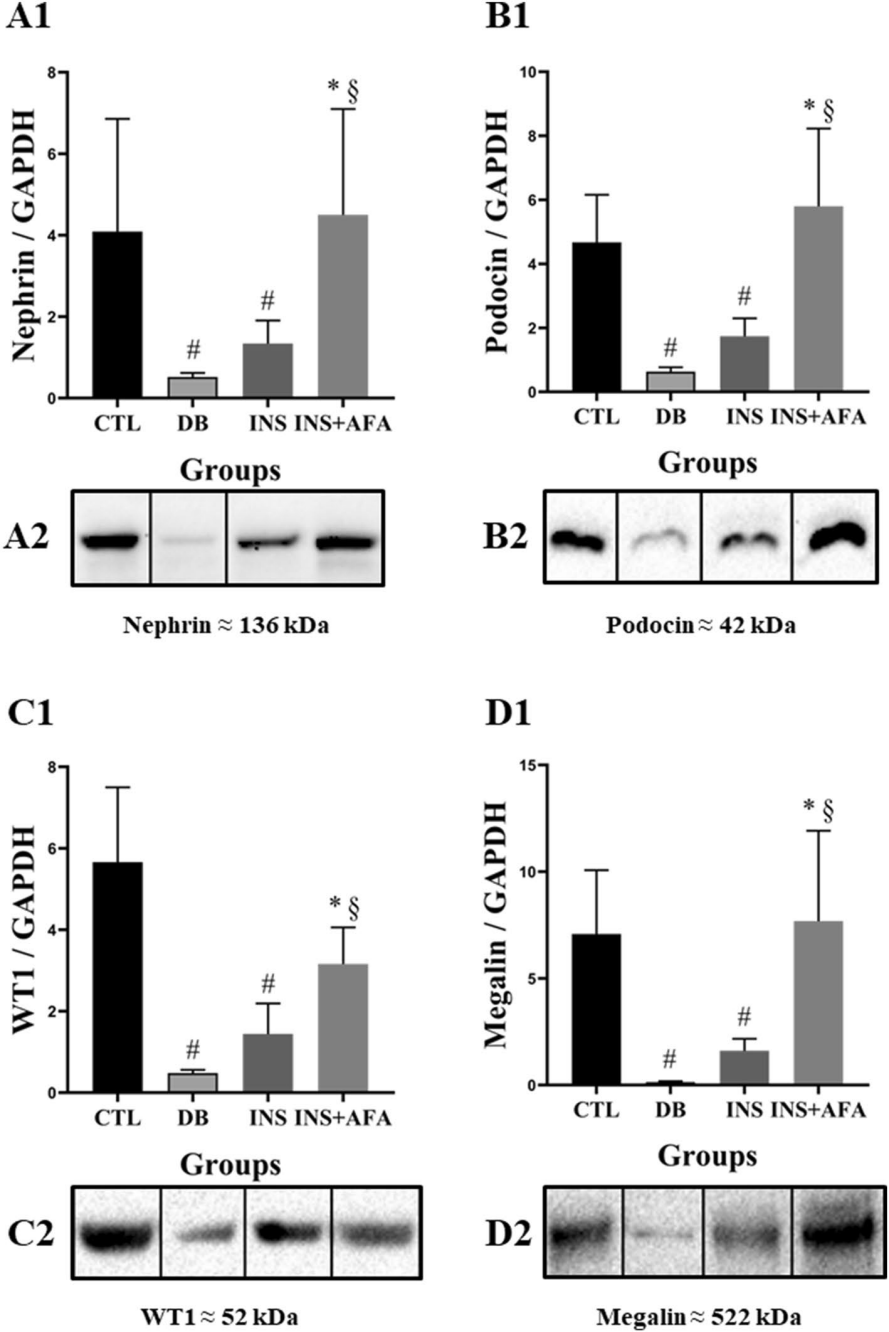
Quantification of protein expression in kidney tissues. Protein densitometric analysis (**A1**; **B1**; **C1**; **D1**); Representative section of western blotting analysis (**A2**; **B2**; **C2**; **D2**). All bands were cropped and full-length blots are shown in Supplementary Figs. 1–4, respectively. Data were normalized to GAPDH expression in the kidney tissue of each animal; CTL, control (n = 11); DB, diabetic (n = 6); INS, insulin (n = 11); INS + AFA, insulin + aqueous extract of *P. edulis* (n = 9); ^#^P < 0.05 vs. control group; *P < 0.05 vs. diabetic group; ^§^P < 0.05 vs. insulin group.

Figure 2 presents protein expression data in the kidney tissue. Compared with the CTL group, the DB group demonstrated a significant reduction in nephrin, podocin, WT1, and megalin expression in the kidney tissue (P = 0.021, P = 0.006, P = 0.004, and P = 0.004, respectively). In addition, the INS group showed a significant reduction in nephrin, podocin, WT1, and megalin expression in the kidney tissue when compared with the CTL group (P = 0.024, P = 0.009, P = 0.006, and P = 0.009, respectively). In contrast, kidney tissue levels of nephrin, podocin, WT1, and megalin protein were higher in the INS + AFA group than in the DB group (P = 0.021, P = 0.009, P = 0.006, and P = 0.009, respectively) and the INS group (P = 0.036, P = 0.009, P = 0.010, and P = 0.011, respectively). Animals in the INS + AFA and CTL groups showed similar kidney expression levels of these proteins.

### Evaluation of urinary protein expression

**Figure 3:**
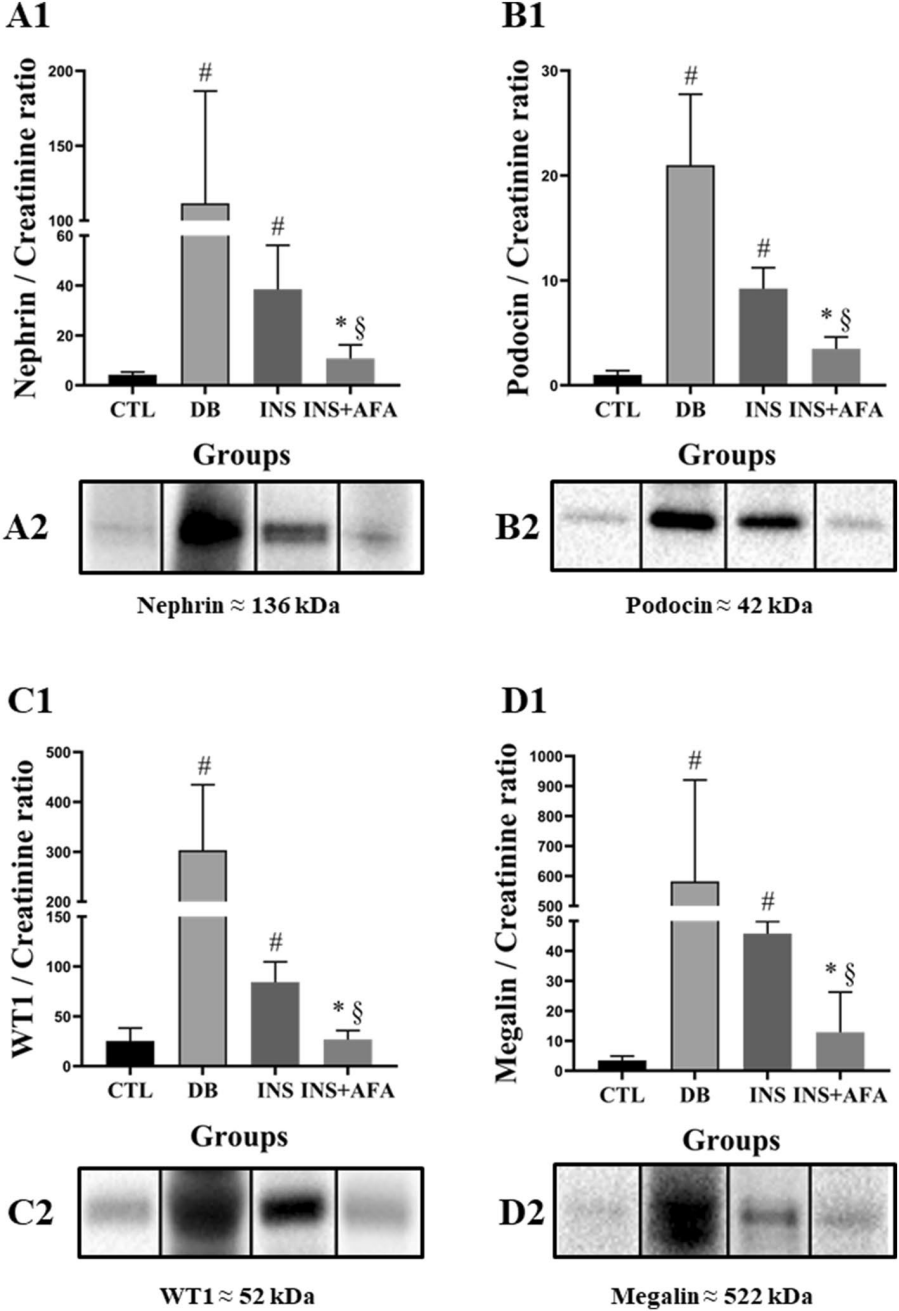
Quantification of urinary protein expression. Protein densitometric analysis (**A1**, **B1**, **C1**,**D1**); Representative section of western blotting analysis (**A2**, **B2**, **C2**, **D2**). All bands were cropped and full-length blots are shown in Supplementary Figs. 21–29, respectively. Data were normalized to GAPDH and then corrected to urinary creatinine of each animal; CTL, control (n = 11); DB, diabetic (n = 6); INS, insulin (n = 11); INS + AFA, insulin + aqueous extract of *P. edulis* (n = 9); ^#^P < 0.05 vs. control group; *P < 0.05 vs. diabetic group; ^§^P < 0.05 vs. insulin group.

Figure 3 presents data on urinary protein expression. Compared with the CTL group, we found a significant increase in urinary nephrin, podocin, WT1, and megalin in the DB group (P = 0.034, P = 0.014, P = 0.004, and P = 0.002, respectively) and INS groups (P = 0.008, P = 0.014, P = 0.004, and P = 0.014, respectively). Furthermore, the INS + AFA group showed a significant reduction in urinary loss of nephrin, podocin, WT1, and megalin when compared with the INS group (P = 0.039, P = 0.020, P = 0.016, and P = 0.020, respectively). The INS + AFA and CTL groups exhibited similar urinary expression levels of these proteins.

Significant positive correlations were noted between urinary podocin and ACR (r = 0.721, P < 0.001) and between urinary WT1 and ACR (r = 0.721, P < 0.001).

### Histopathological evaluation

**Figure 4:**
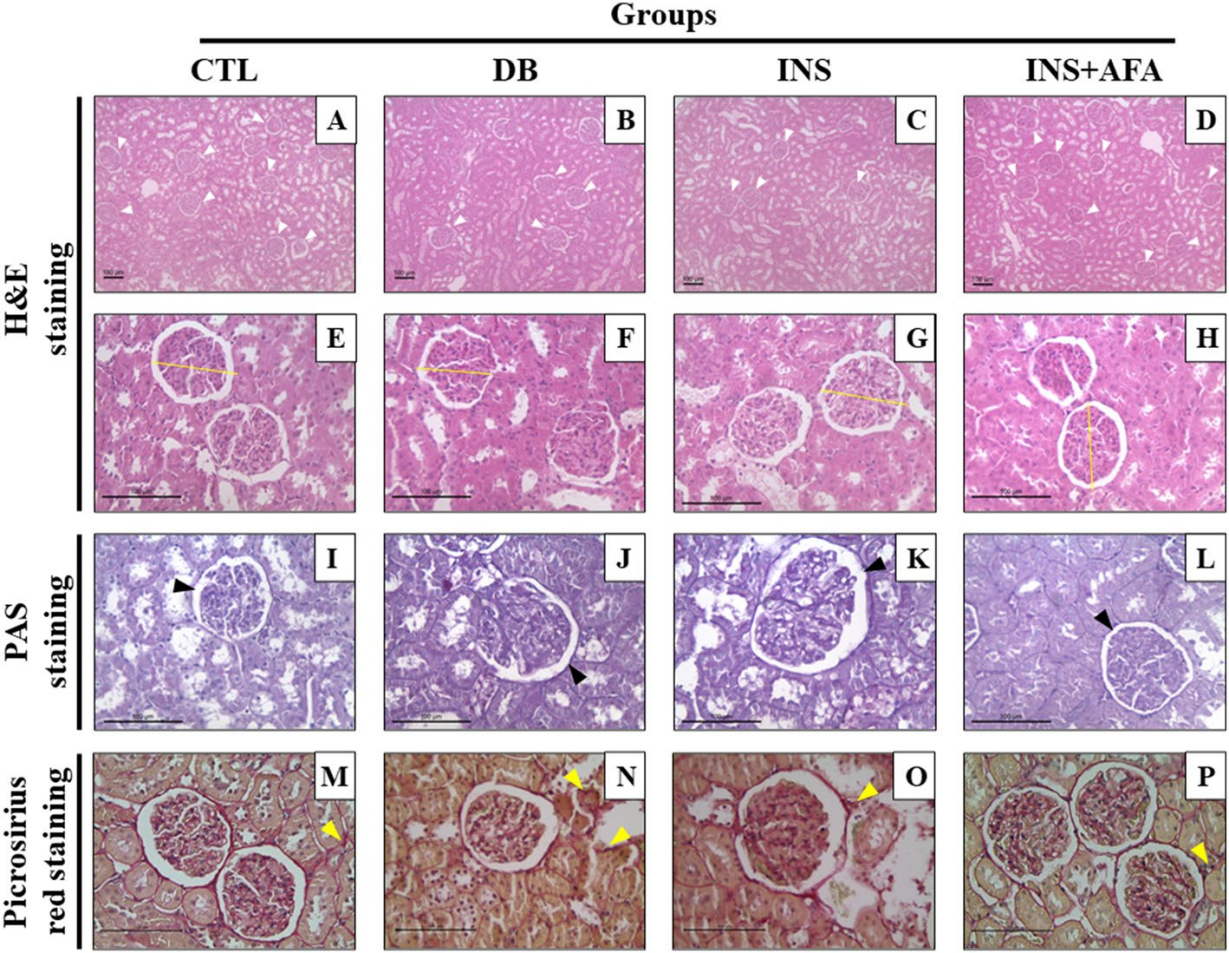
Representative images of histopathological findings. (**A**)–(**D**) Kidney sections stained with hematoxylin and eosin (H&E) at ×100 magnification; white arrowhead = glomerulus. (**E**)–(**H**), kidney sections stained with H&E at ×400 magnification; yellow line = glomerulus’ diameter. (**I**)–(**L**), kidney sections stained with periodic acid–Schiff (PAS) at ×400 magnification; black arrowhead = glomerular basement membrane. (**M**)–(**P**), kidney sections stained with Picrosirius red at ×400 magnification; yellow arrowhead = interstitial fibrosis. CTL, control (n = 11); DB, diabetic (n = 6); INS, insulin (n = 11); INS + AFA, insulin + aqueous extract of *P. edulis* (n = 9).

Table 2 presents the histopathological analyses, and the representative images of histopathological findings are presented in Fig. 4. The number of glomeruli per field was significantly higher in the INS + AFA group than in the INS and DB groups (P < 0.001 for both). As expected, significantly fewer glomeruli per field were noted in the DB group than in the CTL group (P = 0.028). The GBM thickness was significantly reduced in the INS + AFA group when compared with the DB and INS groups (P < 0.001 and P = 0.039, respectively). Conversely, the DB and INS groups showed a significantly thicker GBM than the CTL group (P < 0.001 and P = 0.002, respectively). The number of hyaline bodies was significantly lower in the CTL, INS, and INS + AFA groups than in group DB (P = 0.010, P = 0.011, and P = 0.005, respectively).

**Table 2:** Histological analyses of control, diabetic, and treated diabetic groups.

Variables	Control	Diabetic	Insulin	Insulin + aqueous extract (AFA)
Number of glomeruli, per field	9.90 (9.60–11.00)	9.20 (7.20–9.40)^#^	9.20 (8.80–10.20)	11.40 (11.00–13.00)*^§^
Glomerular diameter, µm	111.90 (106.46–114.49)	117.42 (115.48–119.90)	116.46 (114.01–132.50)	110.79 (103.60–115.31)
Glomerular capillary diameter, μm	94.88 (85.77–99.15)	102.67 (91.60–113.53)	99.88 (96.53–113.17)	101.13 (91.06–101.42)
Bowman's space area, µm	8.00 (7.48–8.59)	8.95 (7.49–11.33)	8.64 (7.52–9.26)	8.52 (7.65–9.53)
Glomerular basement membrane thickness, μm	1.59 (1.48–1.75)	3.61 (3.61–3.70)^#^	2.40 (2.32–2.45)^#^	2.02 (1.68–2.12)*^§^
Hyaline body count, hyaline body number/μm^2^	0.80 (0.20–1.80)	21.80 (4.40–63.80)^#^	1.20 (0.20–5.00)*	0.60 (0.60–1.80)*
Glomerular fibrosis, %	27.99 (26.67–29.23)	32.52 (32.05–32.64)^#^	30.75 (29.69–31.57)^#^	28.50 (27.57–29.19)*^§^
Tubulointerstitial fibrosis, %	7.32 (6.87–7.98)	12.70 (11.42–13.26)^#^	9.05 (8.19–9.84)*	7.21 (6.79–8.14)*^§^

Results are expressed as the median (interquartile range).

^#^P < 0.05, vs. control group; *P < 0.05, diabetic group; ^§^P < 0.05, insulin group.

The glomerular and tubulointerstitial fibrosis rates were significantly higher in the DB group than in the CTL group (P < 0.001 for both). However, the INS + AFA group showed a significant reduction in both parameters when compared with the DB (P < 0.001 for both parameters) and INS groups (P = 0.025 for both parameters). Moreover, we detected a positive correlation between tubulointerstitial fibrosis and urinary megalin levels (r = 0.740, p = 0.036).

## Discussion

Herein, we revealed the nephroprotective action of AFA extract adjuvant to insulin, as indicated by the maintenance of baseline expression levels of mRNA (*Nphs1, Nphs2, Wt1, and Lrp2*) and protein (nephrin, podocin, WT1, and megalin) in the kidney tissue. Interestingly, nephroprotection was also evidenced through the reduction of urinary loss of nephrin, podocin, WT1, and megalin, given that these proteins are markers of kidney glomerular and tubular integrity, and their loss is typically associated with the onset of kidney dysfunction^18–20^.

Nephrin and podocin are slit diaphragm-associated proteins involved in the maintenance of the glomerular filtration barrier. Nephrin, a transmembrane protein with extracellular and intracellular domains, forms the scaffolding of the podocyte slit diaphragm^21^. Podocin, in turn, has a transmembrane domain forming a hairpin structure. The C-terminal cytoplasmic end of podocin interacts with the cytosolic nephrin tail, thereby undergoing oligomerization into lipid rafts to form filtration slits^22^. Thus, alterations in these proteins can impair glomerular filtration and cause kidney dysfunction^23^.

Studies have shown that glomerular and tubular changes are strongly associated with the pathogenesis of DKD^24,25^. In line with this finding, podocyte and tubule biomarkers may predict the onset or progression of DKD^18–20^. Furthermore, accumulated evidence has demonstrated that urinary podocytes and tubular proteins can be positively correlated with hyperglycemia^26,27^, and interestingly, these levels are elevated even in normoalbuminuric patients with diabetes^25,28^. Therefore, nephrin, podocin, WT1, and megalin are more sensitive and specific markers than albuminuria and are valuable for predicting the development and progression of early-stage DKD.

Regarding nephrin and podocin in the present study, we observed that mRNA expression levels of *Nphs1* and *Nphs2* were elevated in the absence of adjuvant treatment with AFA extract, owing to the offset mechanism, induced by the loss of these proteins, to maintain kidney tissue integrity. However, this increase was insufficient to maintain baseline levels of nephrin and podocin in the kidney tissue, and the decreased expression of these proteins may be attributed to the consequent increase in their urinary elimination.

Thus, our data suggest that treatment with AFA extract adjuvant to insulin could prevent the reduced expression of nephrin and podocin in kidney tissue, as well as their urinary loss, given that the respective proteins were expressed at baseline levels necessary to maintain the glomerular filtration barrier.

Furthermore, we observed that AFA extract adjuvant to insulin also exerted a protective action in maintaining WT1 protein in the kidney tissue, as no increase in WT1 levels was detected in the urinary space, indicating possible maintenance of podocyte integrity.

Given that WT1 is a zinc-finger transcription factor that plays an important role in podocyte maturation and maintenance, it is upregulated markedly early and facilitates the transcription of several genes in the kidney tissue, such as *Nphs1* and *Nphs2*, in response to kidney injury^29^. A study by Kalani et al.^6^ has previously highlighted WT1 as an important biomarker of DKD, reporting that WT1 urinary expression is strongly associated with increased proteinuria and decreased renal function in patients with diabetes.

Considering that diabetes can also compromise renal tubular reabsorption, the present study evaluated megalin, a transmembrane protein involved in binding various ligands and abundantly expressed in the apical membranes of the proximal tubule, where it acts by reabsorbing filtered proteins^30^. The absence of megalin leads to proximal tubule dysfunction with tubular proteinuria induced by kidney injury^31^. Our results demonstrated that adjuvant therapy with AFA extract maintained baseline levels of *Lrp2* mRNA expression and megalin protein expression in kidney tissue, preventing urinary loss, indicating a protective effect of the adjuvant treatment on tubular structure and function.

Studies have reported that megalin is also an important early biomarker of DKD, revealing that urinary megalin excretion is linearly associated with ACR and is elevated even in patients with diabetes experiencing earlier-stage DKD^32^.

Furthermore, our results demonstrated that although insulin monotherapy is a classic treatment for diabetes, it was unable to protect renal function, as determined by the significant loss of nephrin, podocin, WT1, and megalin from kidney tissue to the urinary space, reinforcing that adjuvant therapy with *P. edulis* offers a promising alternative for treating diabetes and associated complications.

Interestingly, our histopathological results revealed that the renal microarchitecture was preserved, as determined by the absence of GBM thickening, hyalinization, and glomerular and tubulointerstitial fibrosis in adjuvant therapy-treated animals. These results were reinforced by the maintenance of serum creatinine and ACR values close to those in non-diabetic animals. Literature reports have demonstrated that GBM thickening is an early alteration in diabetes onset, known to increase with disease duration^26,33^. Regarding hyalinization and glomerular and tubulointerstitial fibrosis, literature results demonstrate that they can be correlated with a progressive decline in GFR and tubular reabsorption in DKD^26,34^. Thus, our results demonstrate that AFA extract has a potential role in maintaining kidney tissue integrity.

Although the primary mechanism through which AFA extract can afford nephroprotection in diabetes remains unknown, previous studies performed by partner research groups have demonstrated the adjuvant effect of *P. edulis* fruit peel extracts in terms of glycemic control in diabetic rats^14,15^. These previous studies have suggested that polysaccharides, such as pectin and flavonoids (orientin and isoorientin), are associated with the hypoglycemic effect of the extract and, consequently, prevent the emergence of diabetes complications^14,15^. Additionally, studies have revealed that pectin and other soluble fibers present in the extract can create a layer of gel-like substances in the stomach, delaying the absorption of food and reducing serum glucose^35^. Pectin and flavonoids can also act by modulating the immune response, decreasing the production of pro-inflammatory cytokines, and increasing the production of anti-inflammatory cytokines, which are useful for treating diabetic complications^17,36^.

## Conclusions

The present study suggests that, as an anti-diabetic, AFA extract adjuvant to insulin has potential therapeutic action in reducing and preventing DKD development. This nephroprotection afforded by adjuvant treatment was evidenced by the maintenance of baseline levels of *Nphs1, Nphs2, Wt1*, and *Lrp2* mRNA expression, as well as by preventing the loss of renal proteins such as nephrin, podocin, WT1, and megalin from kidney tissue to the urinary space.

The use of herbal medicinal agents with nephroprotective effects opens new perspectives for adjuvant pharmacological treatment of diabetes. However, further studies are needed to evaluate the in vivo effects and thus support the use of AFA extract as an adjuvant to insulin in anti-diabetic therapy.

## Methods

### Ethical statement

All study protocols, including diabetes induction and euthanasia, were approved by the Ethics Committee on the Use of Animals of the Federal University of Rio Grande do Norte (UFRN) under protocol number 020.019/2017. All methods were performed in accordance with the relevant guidelines and regulations. Animal experiments were conducted while ensuring minimal suffering and limiting the number of specimens needed, according to “the Guide for the Care and Use of Laboratory Animals^37^. All animal experiments in this study are reported in accordance with ARRIVE guidelines.

### Preparation of the AFA extract

The AFA extract was obtained and characterized as described by Cabral et al.^15^. Briefly, seeds and pulp were removed, and peels were dried in an air circulation oven (55 °C) for two days and mechanically triturated to obtain a dry powder (flour)^15^. The flour was extracted with water (1:50, w/v) by decoction for 15 min 100 °C, and solutions were filtered, resulting in an AFA extract^15^.

### Experimental animals

A total of 37 male Wistar rats, aged 5–6 weeks and weighing 220–250 g, were maintained in the animal house of the Health Sciences Center/UFRN under standard conditions with a 12-h light/dark cycle, room temperature of 23 °C ± 1, the humidity of 55% ± 5, throughout the study. Animals were provided food and water ad libitum before and during the experimental period.

### Induction of diabetes

Type 1 diabetes was induced in rats by a single intravenous injection (penile vein) of streptozotocin (STZ) (Sigma-Aldrich, Missouri, USA) dissolved in freshly prepared sodium citrate buffer (0.1 M, pH 4.5) at a dose of 40 mg/kg of body weight^38^. Equal volumes of the vehicle were injected into control rats. Ten days after induction, blood samples were collected from all animals by tail bleeding, and glucose levels were measured using an Accu-Chek Active glucometer (Roche Diagnostics, Basel, Switzerland). Animals exhibiting blood glucose concentrations ≥ 250 mg/dL and clinical signs of polyphagia, polydipsia, polyuria, and loss of body weight were considered diabetic. Treatments were started on day 11 after STZ injection, which was considered day 1 for treatment and continued for 60 days.

### Experimental design

For the experimental study, animals were divided into four groups based on the treatment administered: CTL, non-diabetic rats that did not undergo any intervention (n = 11); DB, diabetes-induced rats that were untreated (n = 6); INS, diabetes-induced rats treated with 10 IU of insulin (n = 11); INS + AFA, diabetes-induced rats treated with 10 IU of insulin and administered 400 mg/kg of AFA extract (n = 9). Insulin-treated animals received once-daily subcutaneous injections of neutral protamine Hagedorn insulin (Novo Nordisk, Bagsværd, Denmark) diluted in 0.9% NaCl (10 IU once a day, at 5 p.m.), and animals treated with AFA extract received once-daily oral doses after insulin administration (at 5 p.m.). Animals were weighed weekly and the dose of AFA extract was corrected for weight.

After 60 days of follow-up and treatment, animals were placed in metabolic cages for 12 h to collect urine samples for biochemical analyses, urinary extracellular vesicle (uEVs) isolation, and protein expression. Subsequently, between 7:00 a.m. and 9:00 a.m., all animals were euthanized with a lethal dose of sodium thiopental (100 mg/kg), and blood samples were obtained by cardiac puncture for biochemical analyses. The kidneys were harvested and stored for subsequent protein and total RNA extraction and histopathological analysis.

### Serum biochemical assessments

Serum glucose, creatinine, total protein, and albumin levels were determined using commercially available kits from Labtest (Labtest Diagnóstica, Minas Gerais, Brazil), in accordance with the manufacturer’s recommendations, using a Labmax Plenno automatic biochemical analyzer (Labtest Diagnóstica).

### Urinary biochemical assessments

Cellular debris from urine samples was removed by centrifugation at 3000×*g* for 5 min. Subsequently, an aliquot was obtained to determine ACR and NGAL levels. Urinary albumin and creatinine concentrations were measured using Labtest kits on a Labmax Plenno automatic biochemical analyzer (Labtest Diagnóstica), following the manufacturer’s recommendations, for subsequent calculation of ACR. NGAL was evaluated using the Lipocalin-2 Rat ELISA Kit (Invitrogen, California, USA), following the manufacturer’s instructions, and read using an Epoch microplate spectrophotometer (BioTek Instruments, Vermont, USA).

### mRNA expression in kidney tissue

The rat kidneys, previously stored at − 80 °C, were macerated, and total RNA was extracted using the SV Total RNA Isolation System kit (Promega, Wisconsin, USA), according to the manufacturer’s instructions. RNA integrity was assessed by capillary electrophoresis performed using an Agilent 2100 Bioanalyzer (Agilent Technologies, California, USA), and samples with an RNA integrity number greater than 8 were accepted. RNA concentrations were measured using a NanoDrop 2000 spectrophotometer (Thermo Scientific, Massachusetts, USA). cDNA synthesis was performed with 1 μg of total RNA using a High-Capacity cDNA Reverse Transcription Kit (Applied Biosystems, Massachusetts, USA), according to the manufacturer’s protocol, using a MyCycler Thermal Cycler (Bio-Rad Laboratories, California, USA). The cDNA was stored at − 20 °C until quantitative reverse transcription-PCR (qRT-PCR) expression assays were performed.

RT-qPCR was performed for the following genes using TaqMan Gene Expression Assays (Applied Biosystems): *Nphs1* (Rn00674268_m1), *Nphs2* (Rn00709834_m1), *Wt1* (Rn00580566_m1), *Lrp2* (Rn00578067_m1), and *Gapdh* (glyceraldehyde-3-phosphate dehydrogenase) (Rn01775763_g1). PCR assays were performed in 96-well plates using a 7500 Fast Real-time PCR System (Applied Biosystems). Relative expression was calculated using the 2^−ΔΔCT^ method^39^, and results are presented as fold-change versus the control group mean values, normalized to *Gapdh.*

### Extraction and isolation of proteins from kidney tissue and uEVs

Proteins from kidney tissue specimens were extracted using RIPA buffer (Sigma-Aldrich, Missouri, USA), according to the manufacturer’s instructions.

For isolating urinary proteins, a protease inhibitor mixture (volume per 30 mL of urine:1.01 mL of 100 mM sodium azide [Sigma-Aldrich, Missouri, USA], 1.2 mL of 10 mM phenylmethylsulfonyl fluoride [Sigma-Aldrich], and 0.03 mL of 2 mM leupeptin [Sigma-Aldrich]) was added to the remaining urine and stored in − 80 °C until analysis. After thawing, the cleared urine supernatant was passed through a 0.20 μm filter and then ultracentrifuged, as described previously^40^. The final supernatant was discarded, and the uEV-containing pellet was dissolved in Laemmli buffer (Bio-Rad Laboratories, California, USA) for protein expression.

The protein concentration in the kidney tissue and uEVs was assessed using the Pierce 660 nm Protein Assay Kit (Invitrogen, California, USA), according to the manufacturer’s protocol.

### Protein expression in kidney tissues and uEVs

Proteins (50 µg/ml were loaded into each well) from the kidney tissue and uEVs were separated by one-dimensional gel electrophoresis using NuPAGE 4–12% Bis–Tris gel (Invitrogen, California, USA). Subsequently, the proteins were transferred to polyvinylidene difluoride (PVDF) membranes (iBlot Transfer Stack, Invitrogen) using the iBlot Dry Blotting Transfer System (Invitrogen). Western blot analysis was performed using the following antibodies: rabbit monoclonal nephrin (ab216341; Abcam Plc, Cambridge, UK), rabbit monoclonal podocin (ab181143; Abcam Plc), rabbit polyclonal LRP2 (megalin) (PA5-67900; Invitrogen), rabbit polyclonal GAPDH (glyceraldehyde-3-phosphate dehydrogenase) (PA1-987; Invitrogen), horseradish peroxidase-conjugated goat anti-rabbit IgG (ab97051; Abcam Plc), mouse monoclonal WT1 (sc-393498; Santa Cruz Biotechnology, Texas, USA), and horseradish peroxidase-conjugated goat anti-mouse IgG (sc-2005; Santa Cruz Biotechnology). Non-specific antibody binding was reduced by incubating membranes with 5% non-fat dry milk. The antibody-antigen reaction was visualized after exposure to Amersham ECL Prime western blotting Detection Reagent (GE Healthcare, Uppsala, Sweden), and images were captured using a ChemiDoc image analyzer (Bio-Rad Laboratories, California, USA). Densitometric analysis of nephrin, podocin, WT1, and megalin bands was performed using Image Lab Software (Bio-Rad Laboratories) and normalized to GAPDH expression. The values of nephrin, podocin, WT1, and megalin densitometric analysis obtained from uEVs were subsequently corrected using urinary creatinine concentrations^41,42^.

### Histopathological study

Histopathological studies were performed using kidney sections fixed in 10% formalin solution, embedded in paraffin, and sectioned at 5 μm. The tissue sections were then deparaffinized, rehydrated, mounted on glass slides, and stained with hematoxylin and eosin (H&E) (to detect changes in cellular morphology), Picro-Sirius red (to detect collagen deposition), and periodic acid–Schiff (PAS) (to detect polysaccharides). Subsequently, the sections were examined under a DM500 microscope with an ICC50 camera attached (Leica Microsystems, Wetzlar, Germany) at 100× and 400× magnifications. Five images from five random fields were captured at each magnification for each stain. Results were quantified using ImageJ software 1.8v (National Institutes of Health, Maryland, USA).

### Statistical analyses

The distribution of continuous variables was assessed using the Shapiro–Wilk test, and the normality test failed for all data. Nonparametric data were analyzed using the Kruskal–Wallis test followed by the Mann–Whitney test and correlated using Spearman’s rank correlation test. Assessments were performed using SPSS Statistics (version 20.0; IBM, New York, USA) and GraphPad PRISM version 5.0; GraphPad Software Inc., California, USA). Statistical significance was set at P < 0.05.

## Supplementary Information

Supplementary Figures.

## Data Availability

The datasets generated and/or analyzed during the current study are available from the corresponding author upon request.
