# Supplementary material for: The nephroprotective action of Passiflora edulis in streptozotocin-induced diabetes
Source: Sci Rep. 2022 Oct 20;12:17546. doi: 10.1038/s41598-022-21826-9 (PMC9584925; doi:10.1038/s41598-022-21826-9)
Supplement: Supplementary file 1 — Supplementary Figures. [file 41598_2022_21826_MOESM1_ESM.pdf]

The nephroprotective action of *Passiflora edulis* in streptozotocin-induced diabetes

Ony Araújo Galdino[1], Iago de Souza Gomes[1], Renato Ferreira de Almeida Júnior[1], Maria Imaculada Conceição Ferreira de Carvalho[1], Bento João Abreu[2], Marcela Abbott Galvão Ururahy[1], Barbara Cabral[3], Silvana Maria Zucolotto Langassner[3], Karla Simone Costa de Souza[1], Adriana Augusto de Rezende[1*].

[1] Department of Clinical and Toxicological Analyses, Federal University of Rio Grande do Norte, Natal, RN, Brazil;

[2] Department of Morphology, Federal University of Rio Grande do Norte, Natal, RN, Brazil;

[3] Department of Pharmacy, Federal University of Rio Grande do Norte, Natal, RN, Brazil.

* Corresponding author:

Adriana Augusto de Rezende

Av. General Gustavo Cordeiro de Farias, S/N, Faculty of Pharmacy, Petrópolis, CEP: 59012-570, Natal, RN, Brazil

e-mail: adrirezende@yahoo.com

Original blot images (Figure 2 - Protein expression in kidney tissues)

Red boxes in figures 01-04 represent areas from which representative bands were cropped for figures A2; B2; C2; D2.

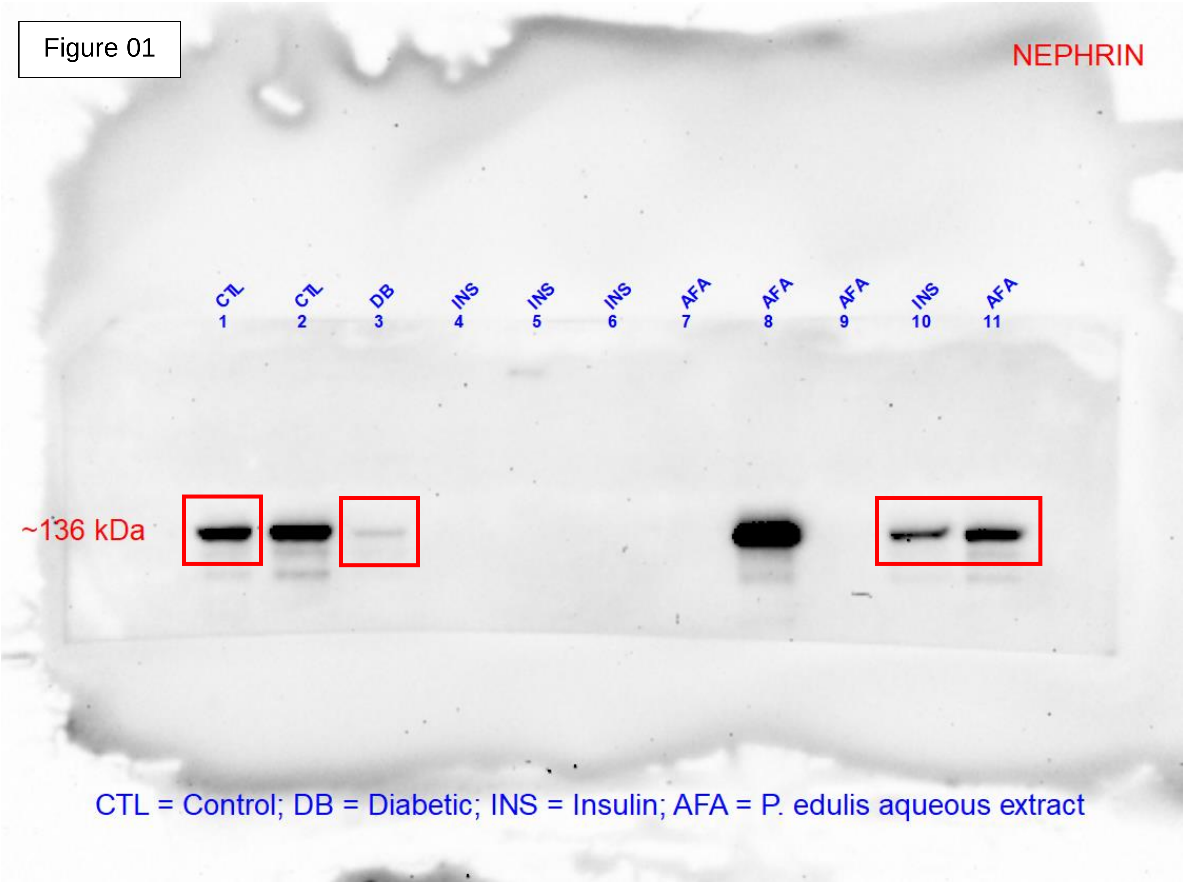

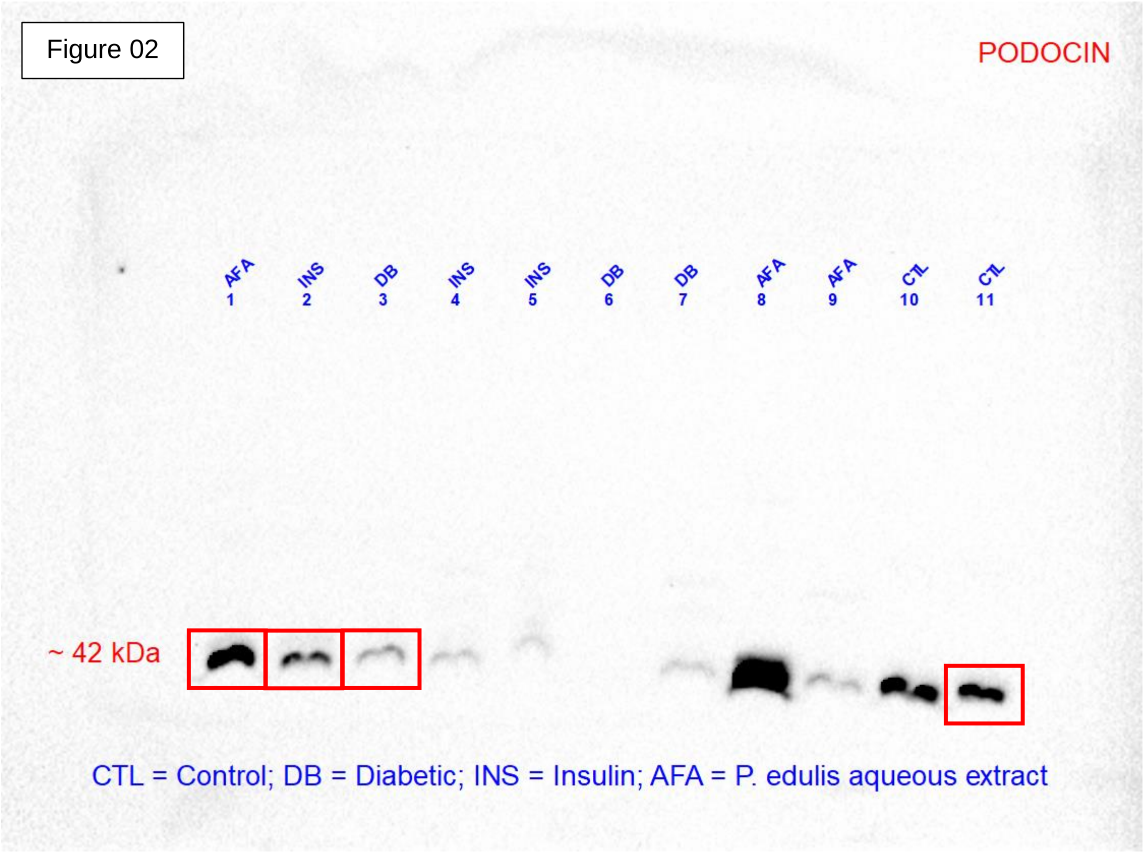

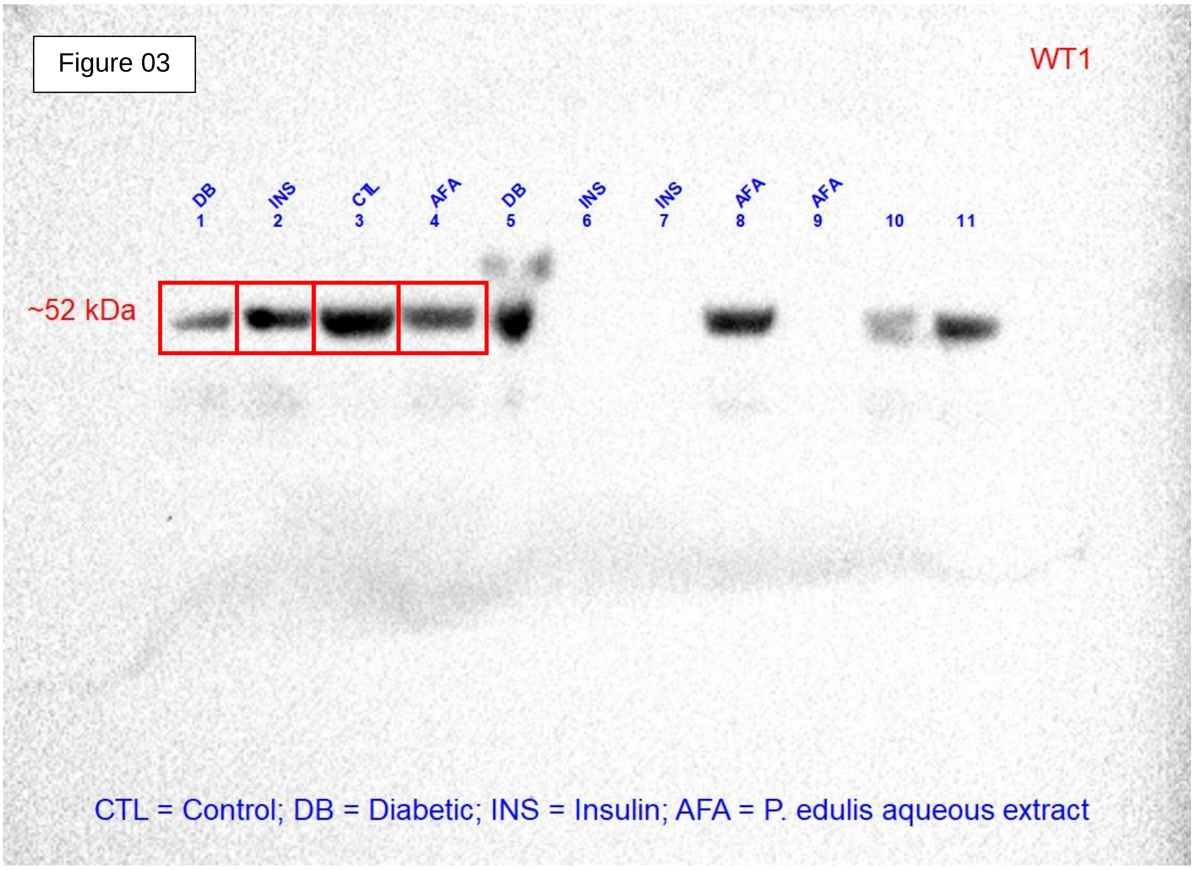
Figure 03
WT1
DB
1
INS
2
CTL
3
AFA
4
DB
5
INS
6
INS
7
AFA
8
AFA
9
10
11
~52 kDa
CTL = Control; DB = Diabetic; INS = Insulin; AFA = P. edulis aqueous extract

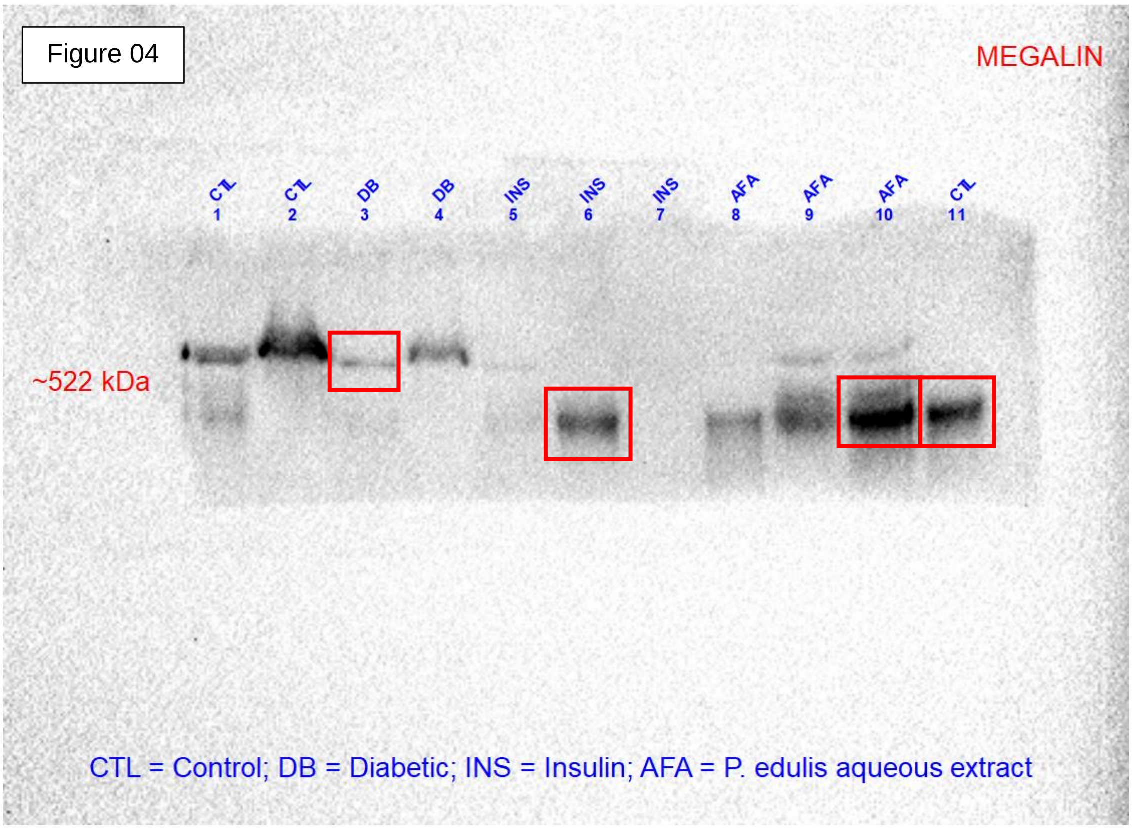
Figure 04
MEGALIN
CTL
1
CTL
2
DB
3
DB
4
INS
5
INS
6
INS
7
AFA
8
AFA
9
AFA
10
CTL
11
~522 kDa
CTL = Control; DB = Diabetic; INS = Insulin; AFA = P. edulis aqueous extract

Figure 05

GAPDH

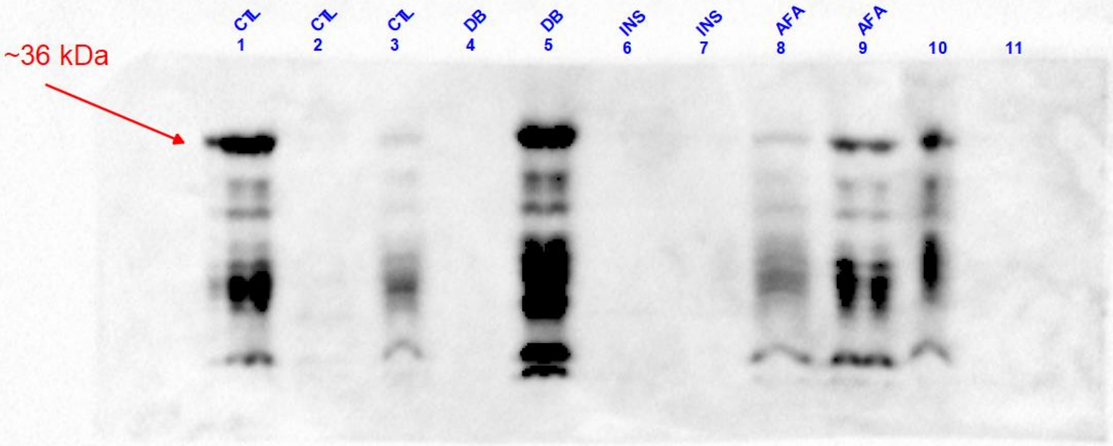

CTL = Control; DB = Diabetic; INS = Insulin; AFA = P. edulis aqueous extract

Figure 06

MEGALIN

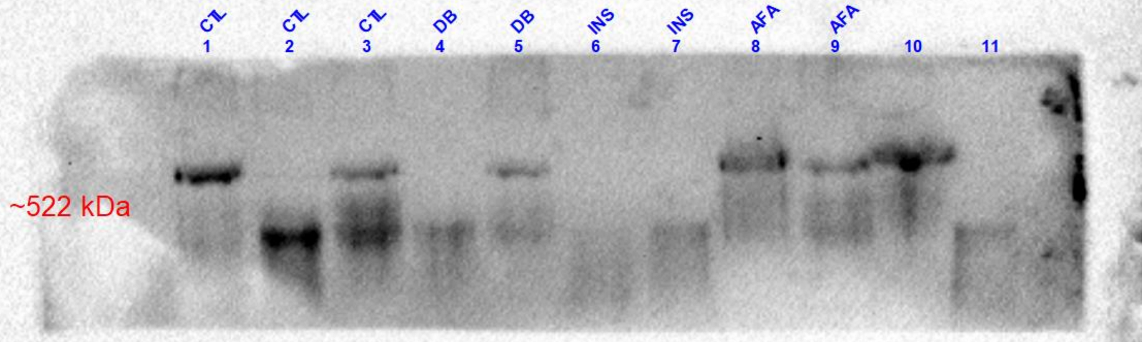

CTL = Control; DB = Diabetic; INS = Insulin; AFA = P. edulis aqueous extract

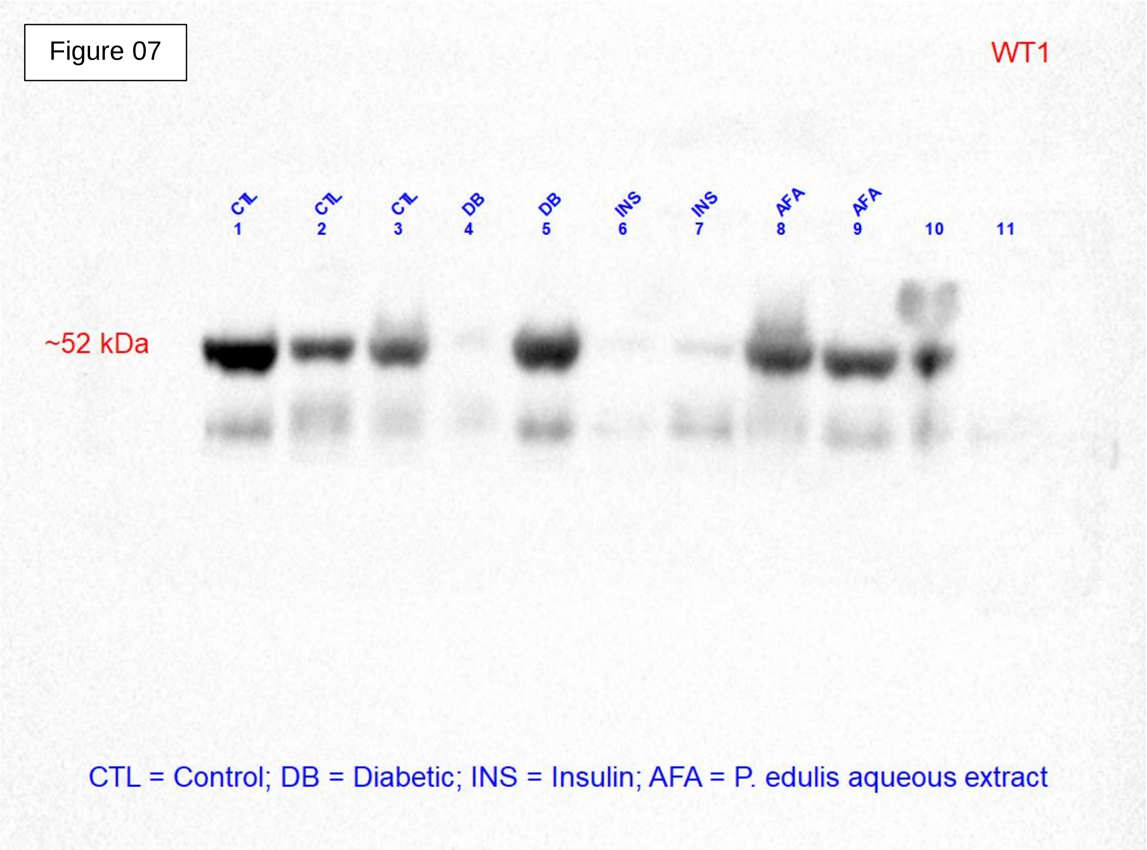
Figure 07
WT1
CTL 1
CTL 2
CTL 3
DB 4
DB 5
INS 6
INS 7
AFA 8
AFA 9
10
11
~52 kDa
CTL = Control; DB = Diabetic; INS = Insulin; AFA = P. edulis aqueous extract

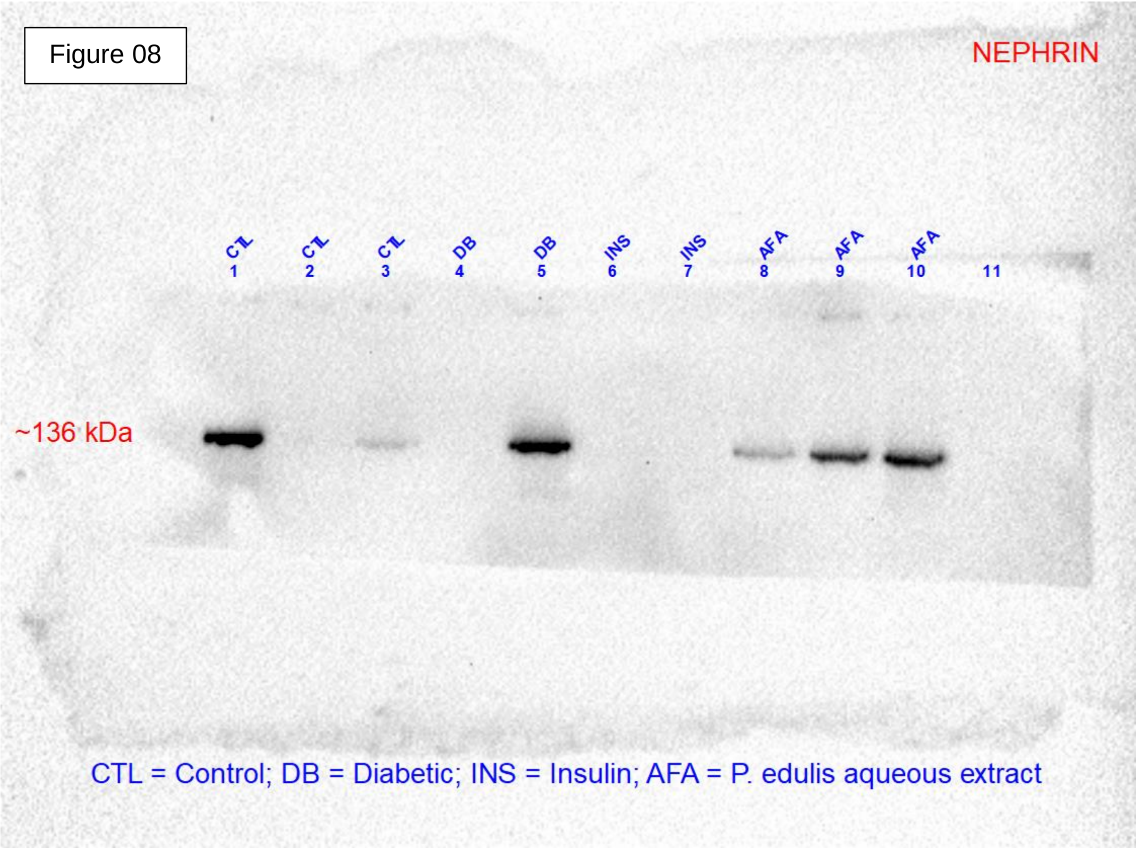
Figure 08
NEPHRIN
CTL 1
CTL 2
CTL 3
DB 4
DB 5
INS 6
INS 7
AFA 8
AFA 9
AFA 10
11
~136 kDa
CTL = Control; DB = Diabetic; INS = Insulin; AFA = P. edulis aqueous extract

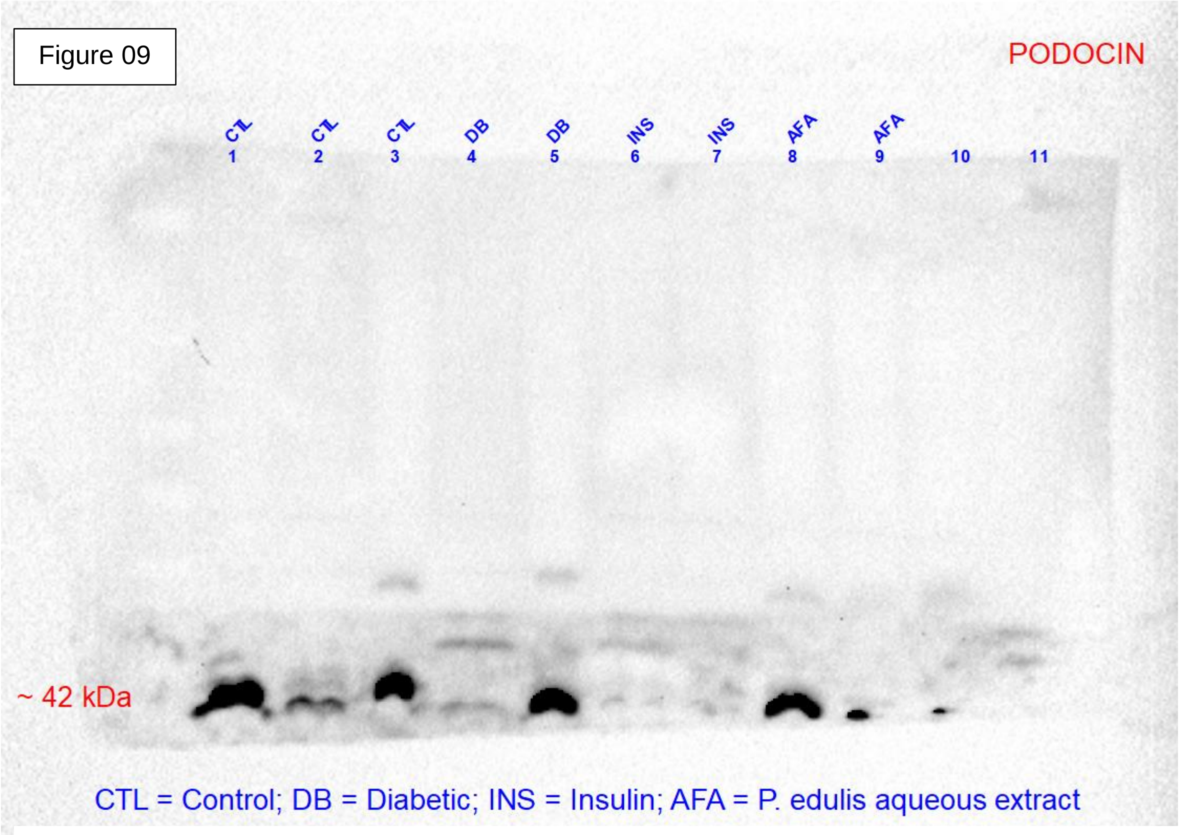
Figure 09
PODOCIN
CTL 1
CTL 2
CTL 3
DB 4
DB 5
INS 6
INS 7
AFA 8
AFA 9
10
11
~ 42 kDa
CTL = Control; DB = Diabetic; INS = Insulin; AFA = P. edulis aqueous extract

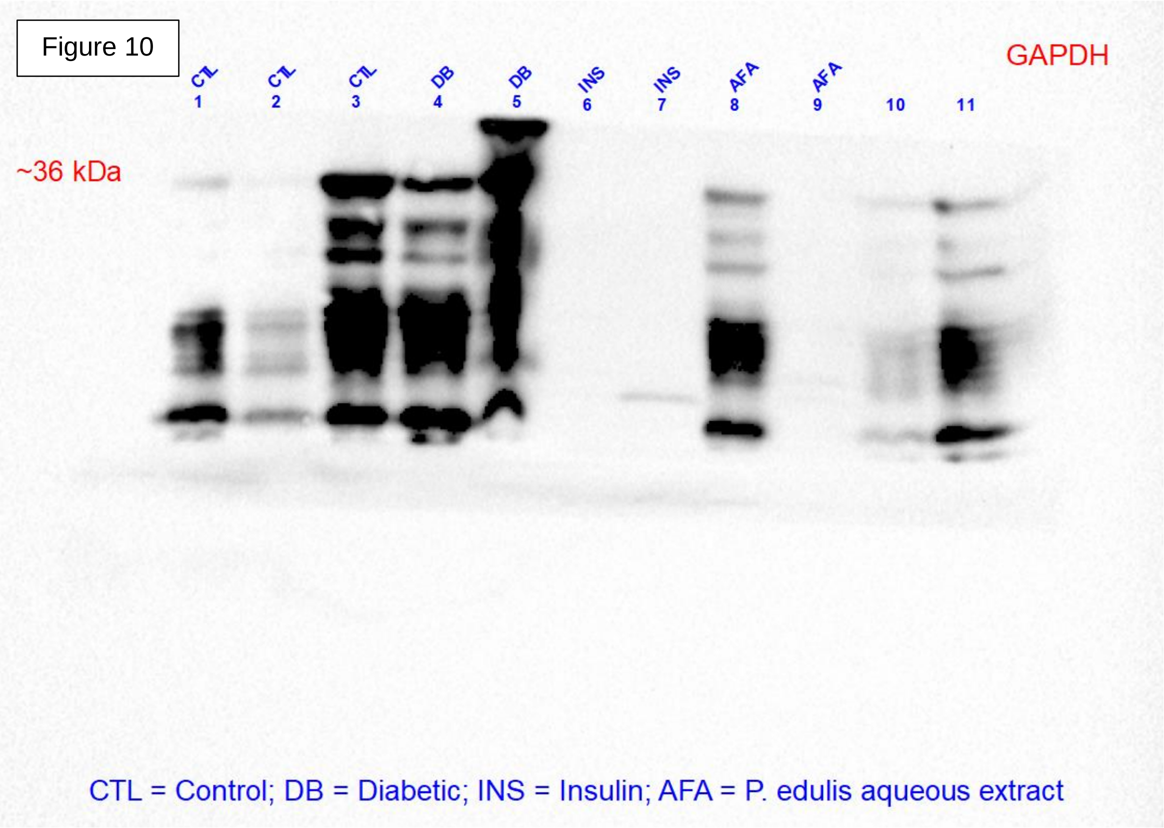
Figure 10
GAPDH
CTL 1
CTL 2
CTL 3
DB 4
DB 5
INS 6
INS 7
AFA 8
AFA 9
10
11
~36 kDa
CTL = Control; DB = Diabetic; INS = Insulin; AFA = P. edulis aqueous extract

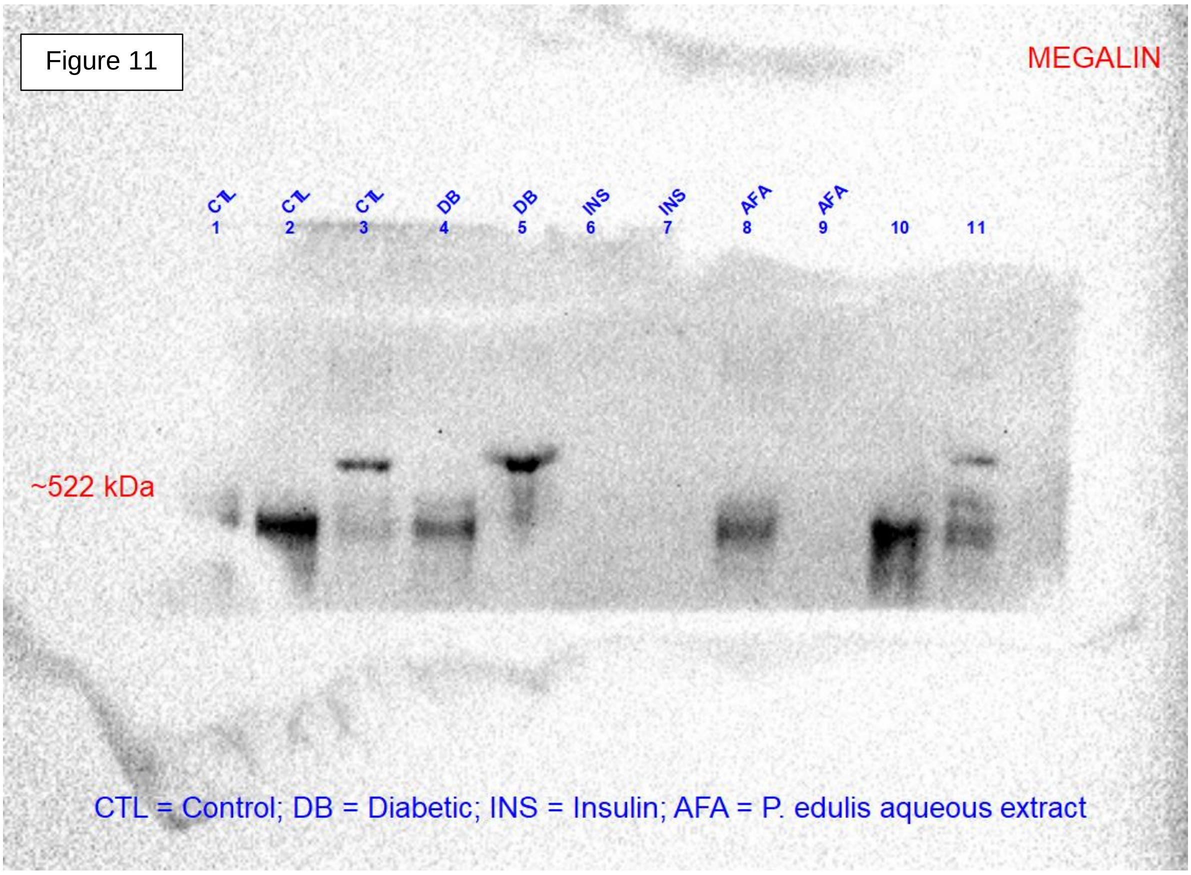
Figure 11
MEGALIN
CTL CTL CTL DB DB INS INS AFA AFA
1 2 3 4 5 6 7 8 9 10 11
~522 kDa
CTL = Control; DB = Diabetic; INS = Insulin; AFA = P. edulis aqueous extract

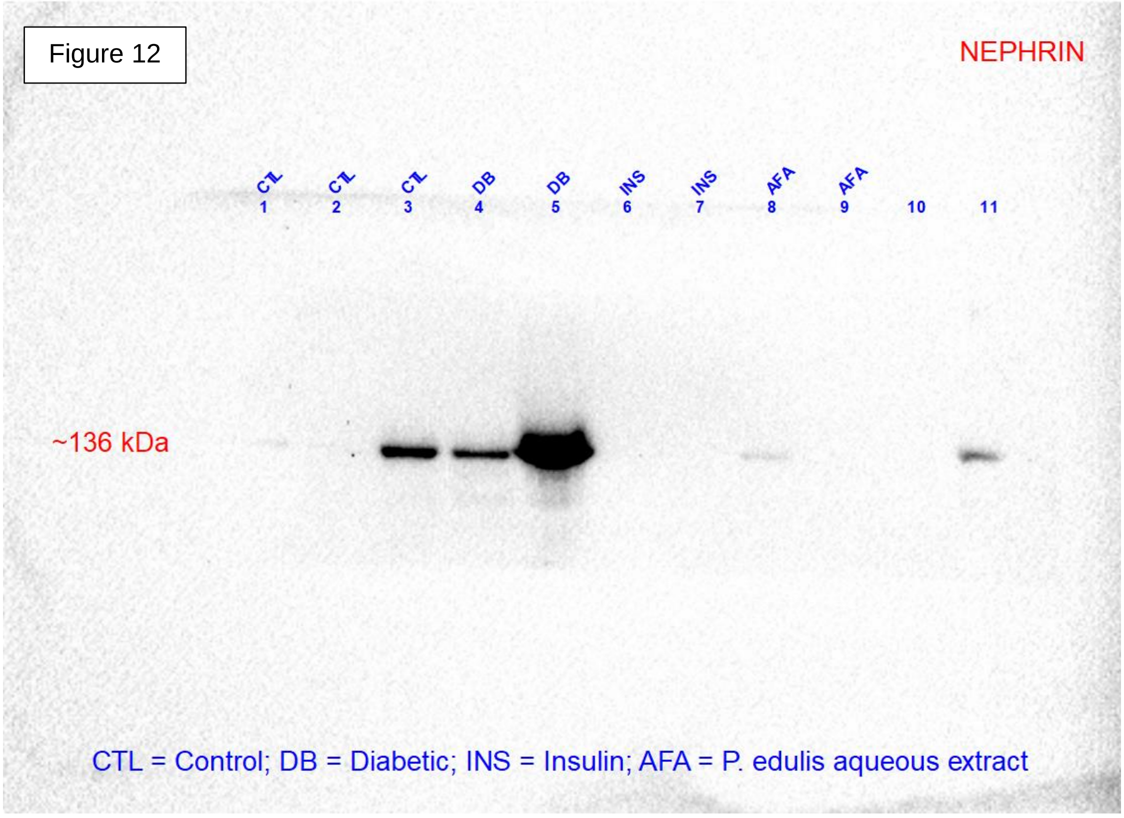
Figure 12
NEPHRIN
CTL CTL CTL DB DB INS INS AFA AFA
1 2 3 4 5 6 7 8 9 10 11
~136 kDa
CTL = Control; DB = Diabetic; INS = Insulin; AFA = P. edulis aqueous extract

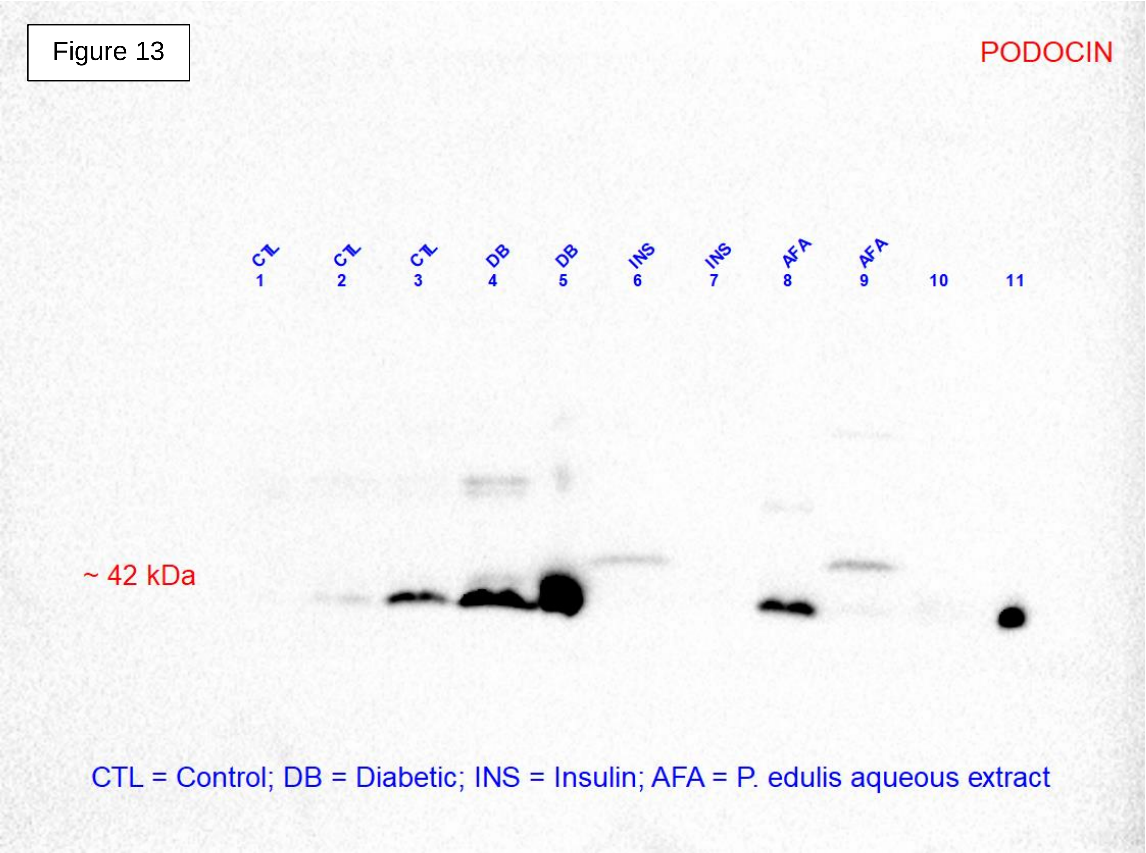
Figure 13
PODOCIN
CTL 1
CTL 2
CTL 3
DB 4
DB 5
INS 6
INS 7
AFA 8
AFA 9
10
11
~ 42 kDa
CTL = Control; DB = Diabetic; INS = Insulin; AFA = P. edulis aqueous extract

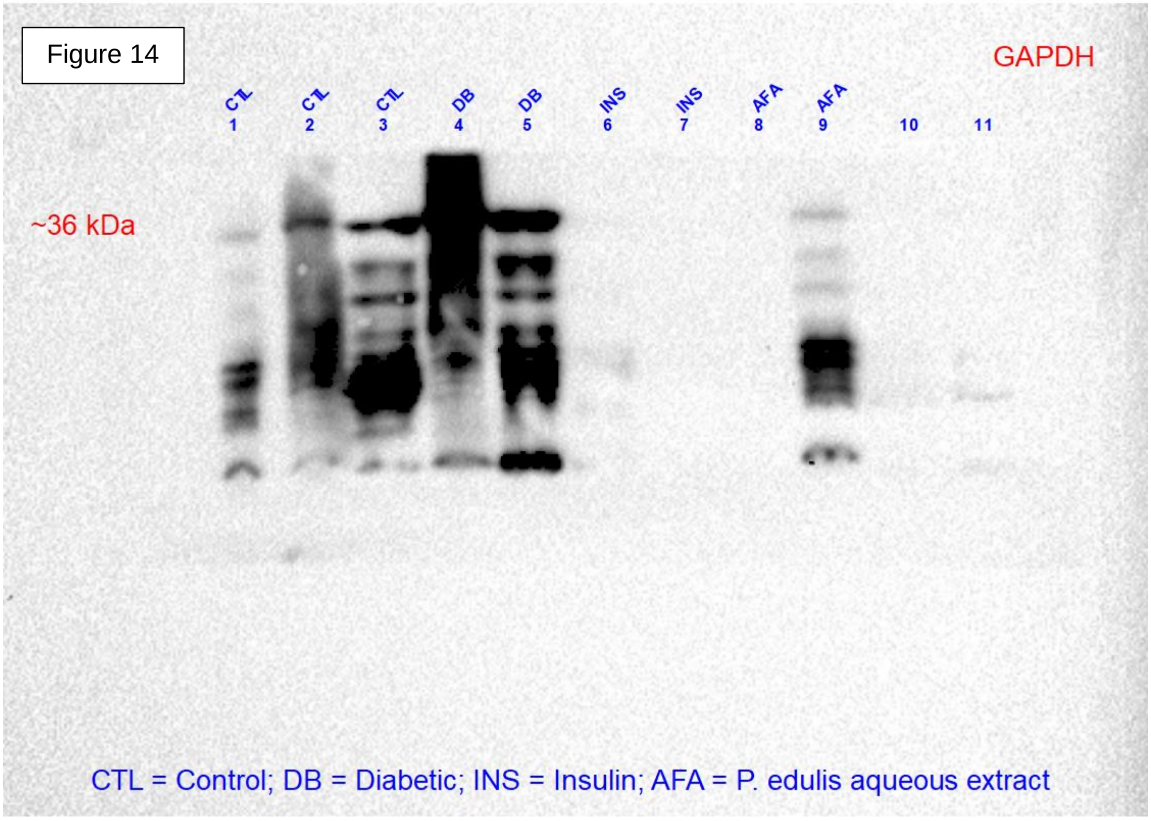
Figure 14
GAPDH
CTL 1
CTL 2
CTL 3
DB 4
DB 5
INS 6
INS 7
AFA 8
AFA 9
10
11
~36 kDa
CTL = Control; DB = Diabetic; INS = Insulin; AFA = P. edulis aqueous extract

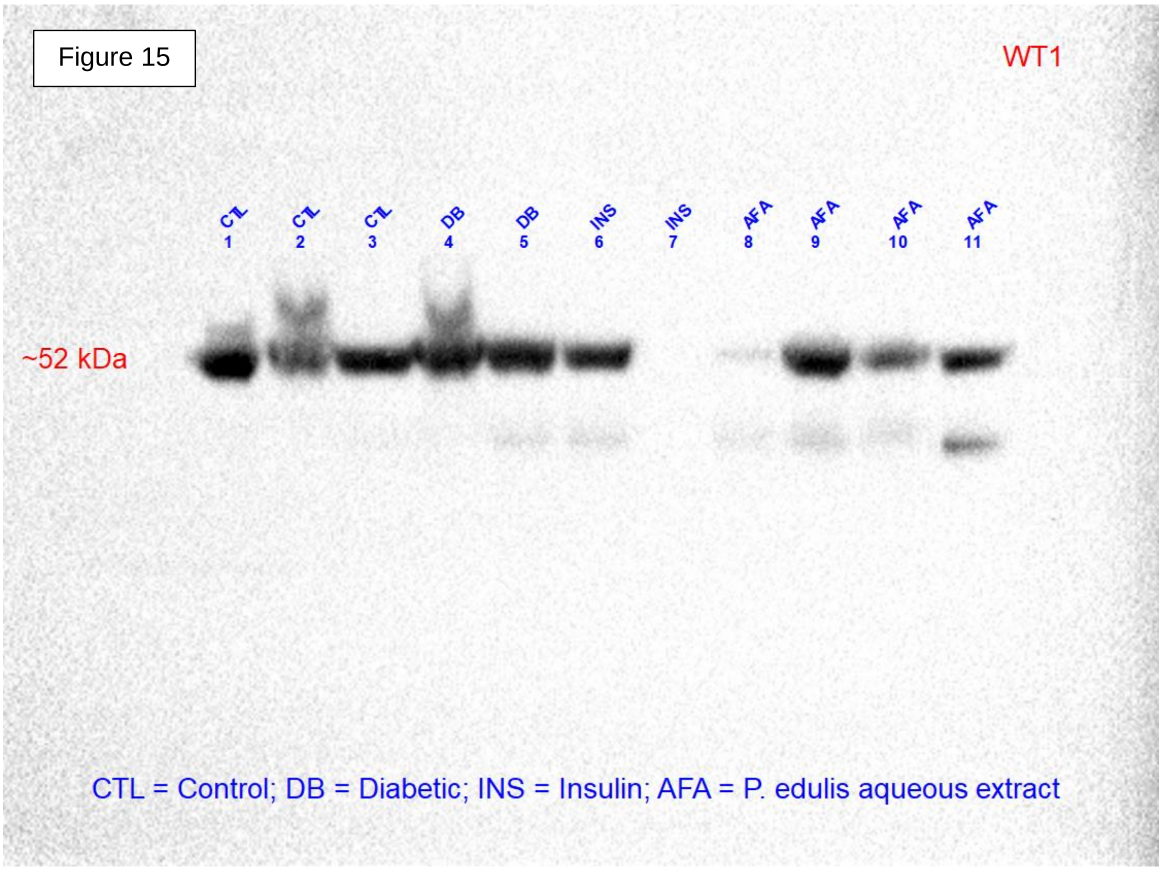
Figure 15
WT1
CTL 1
CTL 2
CTL 3
DB 4
DB 5
INS 6
INS 7
AFA 8
AFA 9
AFA 10
AFA 11
~52 kDa
CTL = Control; DB = Diabetic; INS = Insulin; AFA = P. edulis aqueous extract

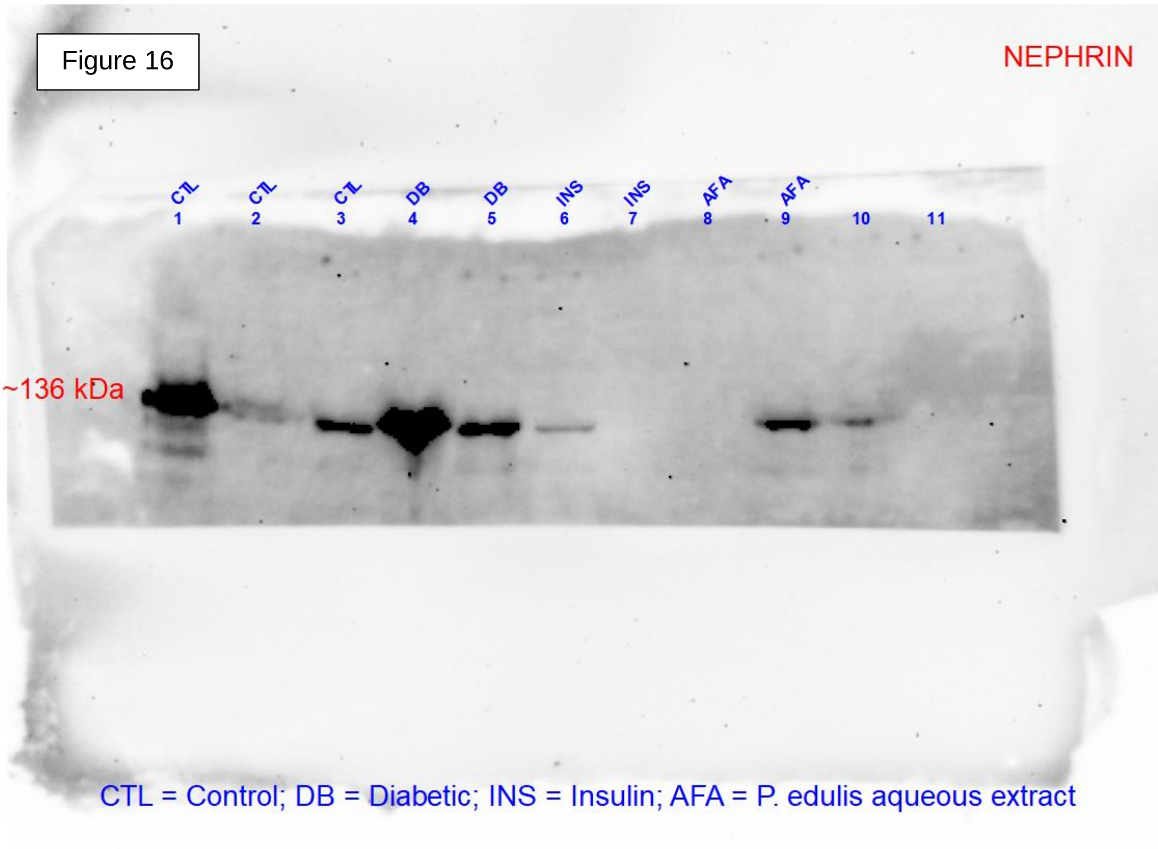
Figure 16
NEPHRIN
CTL 1
CTL 2
CTL 3
DB 4
DB 5
INS 6
INS 7
AFA 8
AFA 9
10
11
~136 kDa
CTL = Control; DB = Diabetic; INS = Insulin; AFA = P. edulis aqueous extract

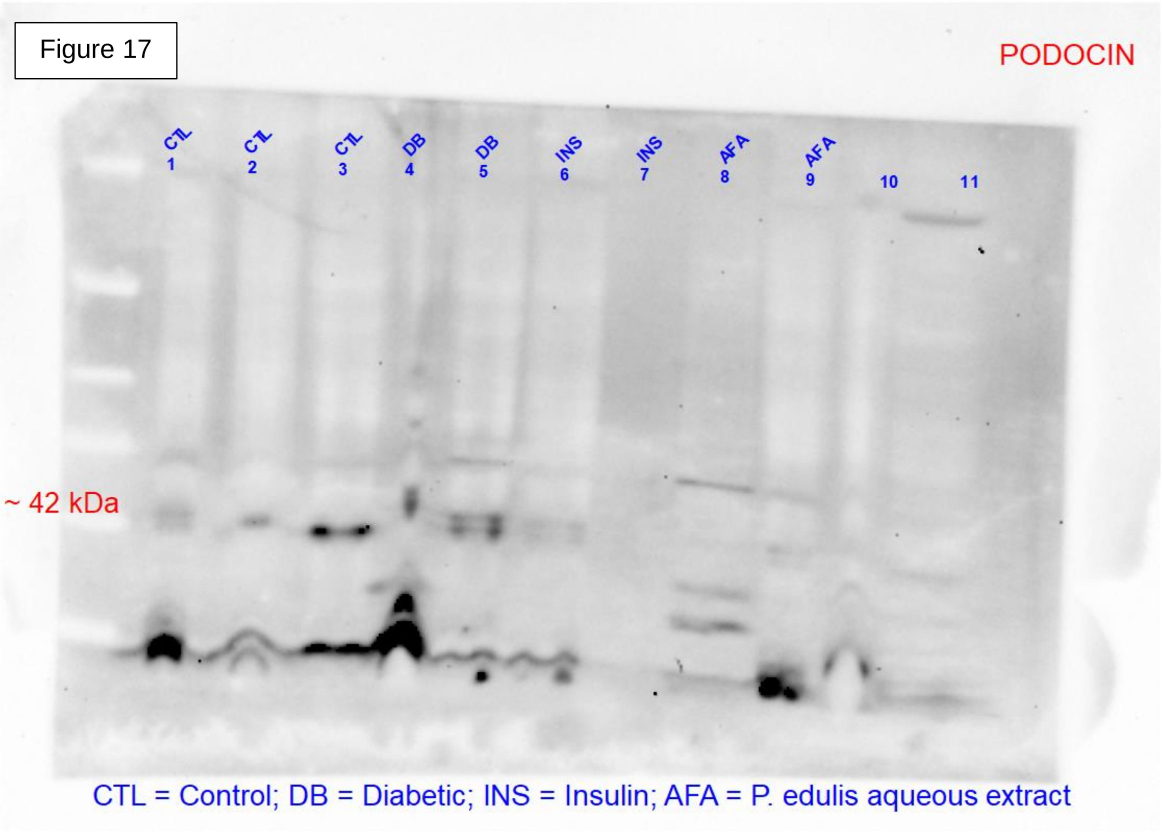
Figure 17
PODOCIN
CTL 1
CTL 2
CTL 3
DB 4
DB 5
INS 6
INS 7
AFA 8
AFA 9
10
11
~ 42 kDa
CTL = Control; DB = Diabetic; INS = Insulin; AFA = P. edulis aqueous extract

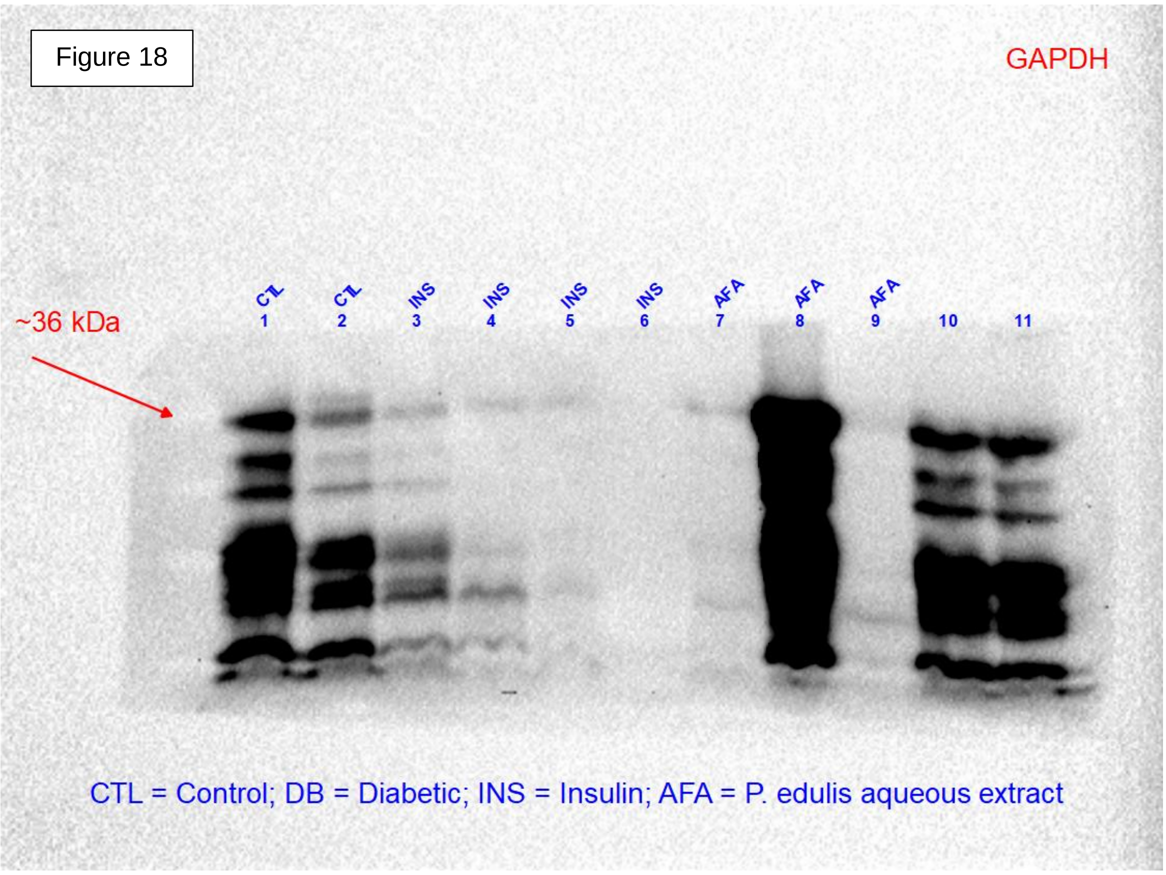
Figure 18
GAPDH
~36 kDa
CTL 1
CTL 2
INS 3
INS 4
INS 5
INS 6
AFA 7
AFA 8
AFA 9
10
11
CTL = Control; DB = Diabetic; INS = Insulin; AFA = P. edulis aqueous extract

Figure 19

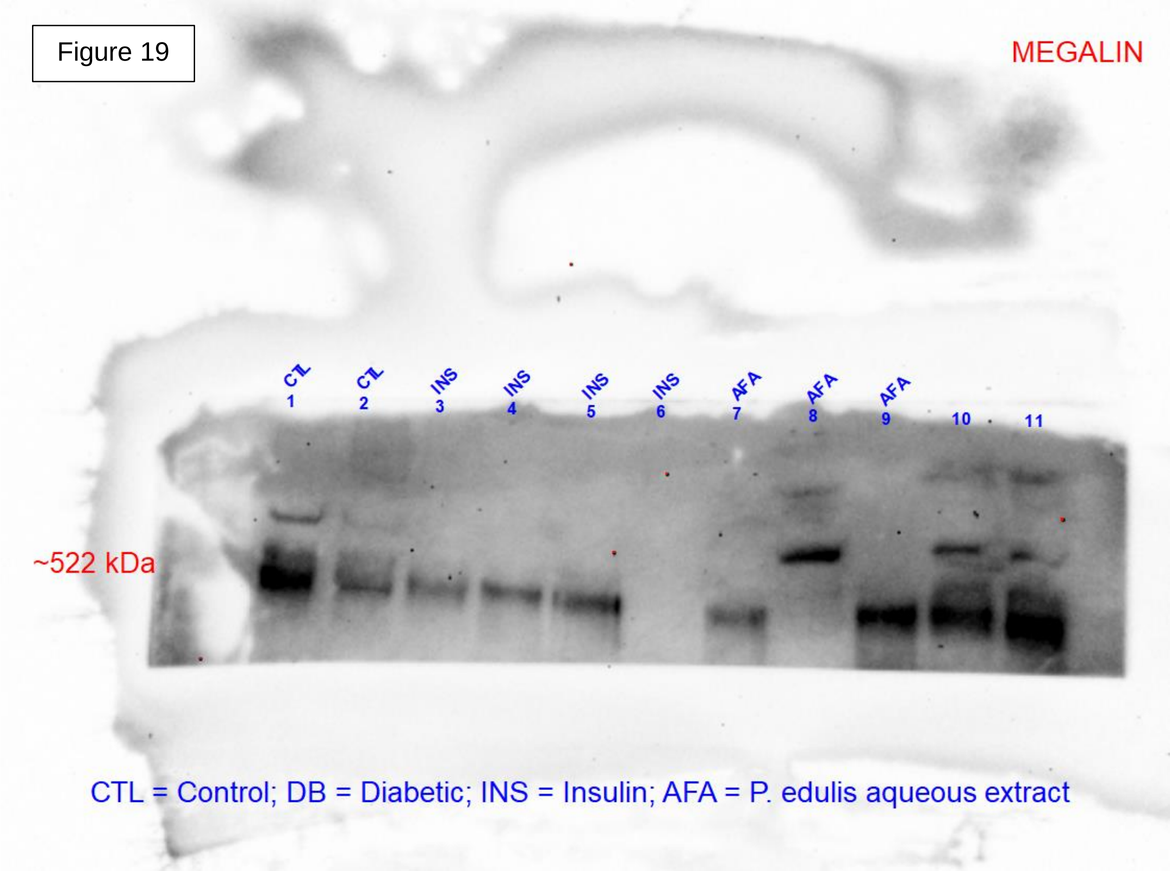

Figure 20

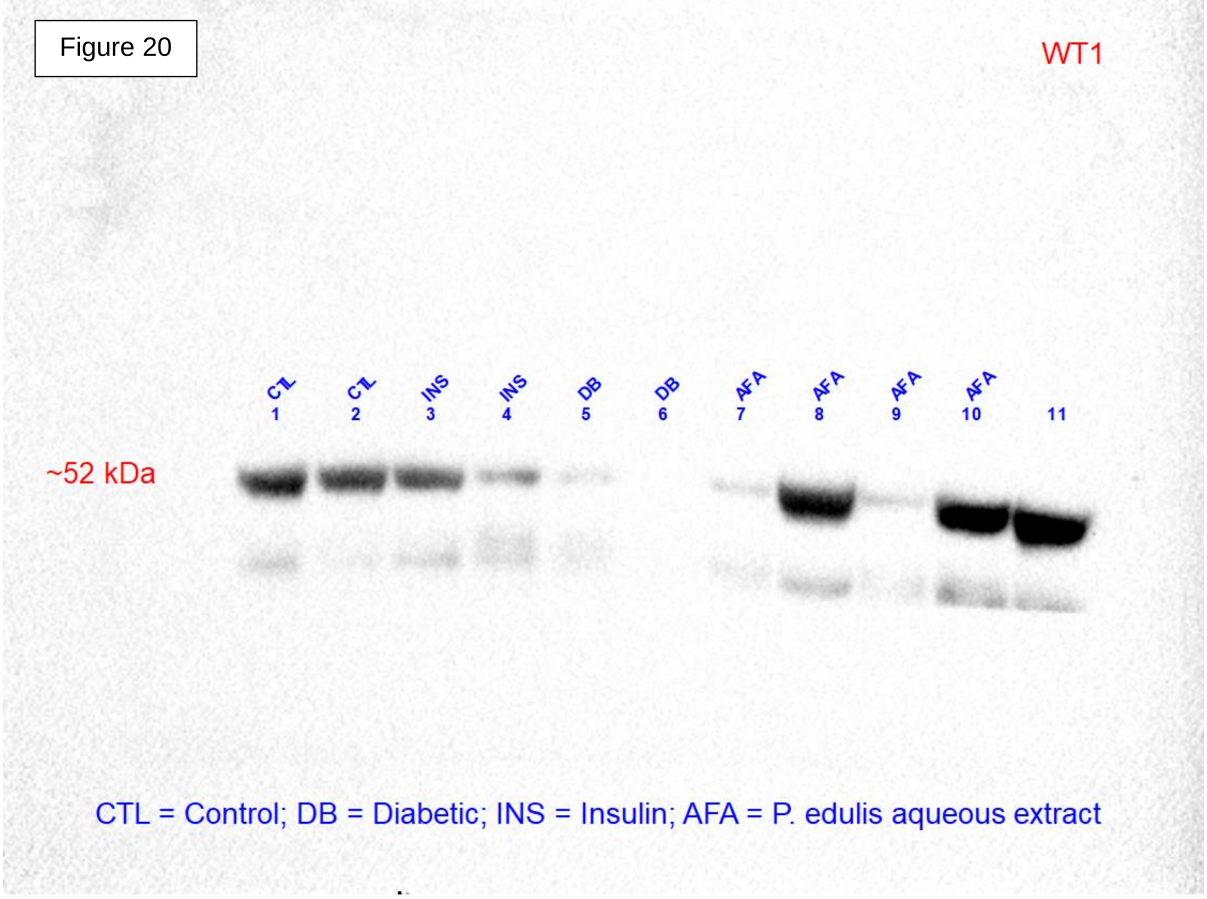

Original blot images (Figure 3 - Urinary protein expression)

Red boxes in figures 21-29 represent areas from which representative bands were cropped for figures A2; B2; C2; D2.

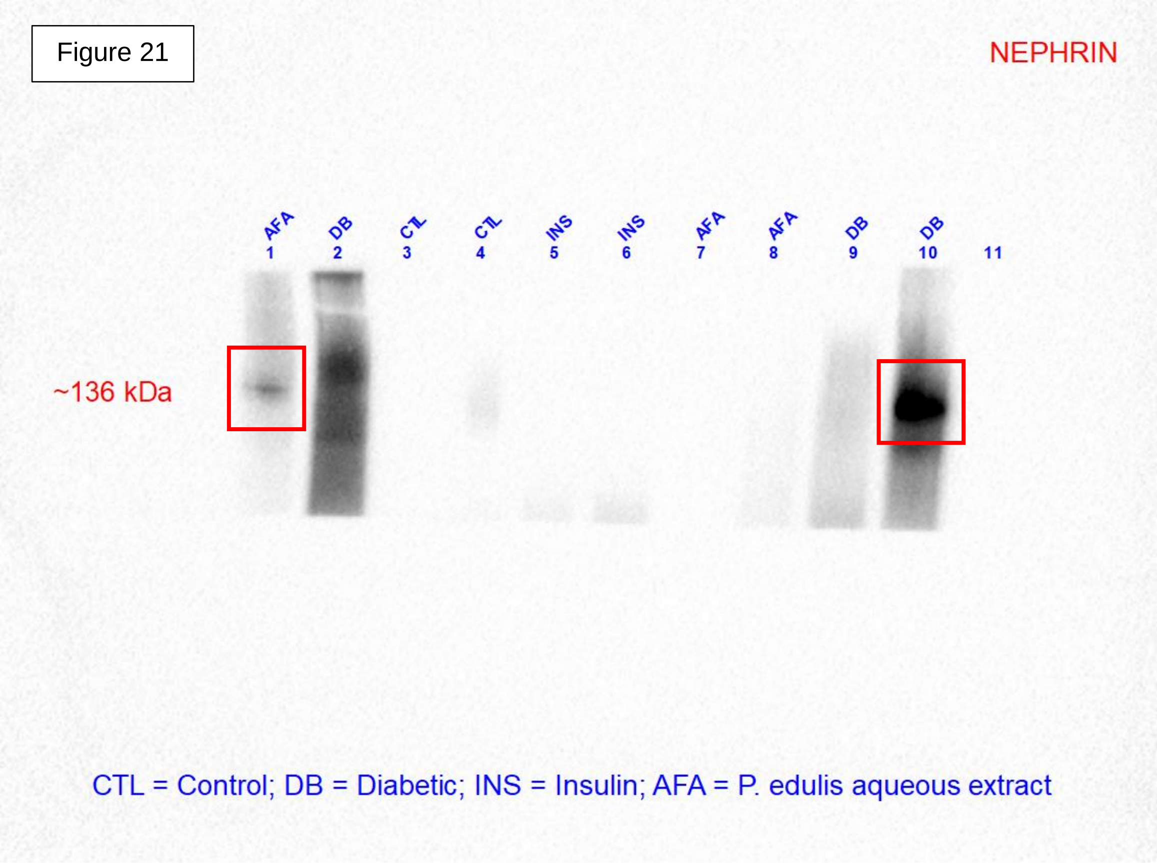

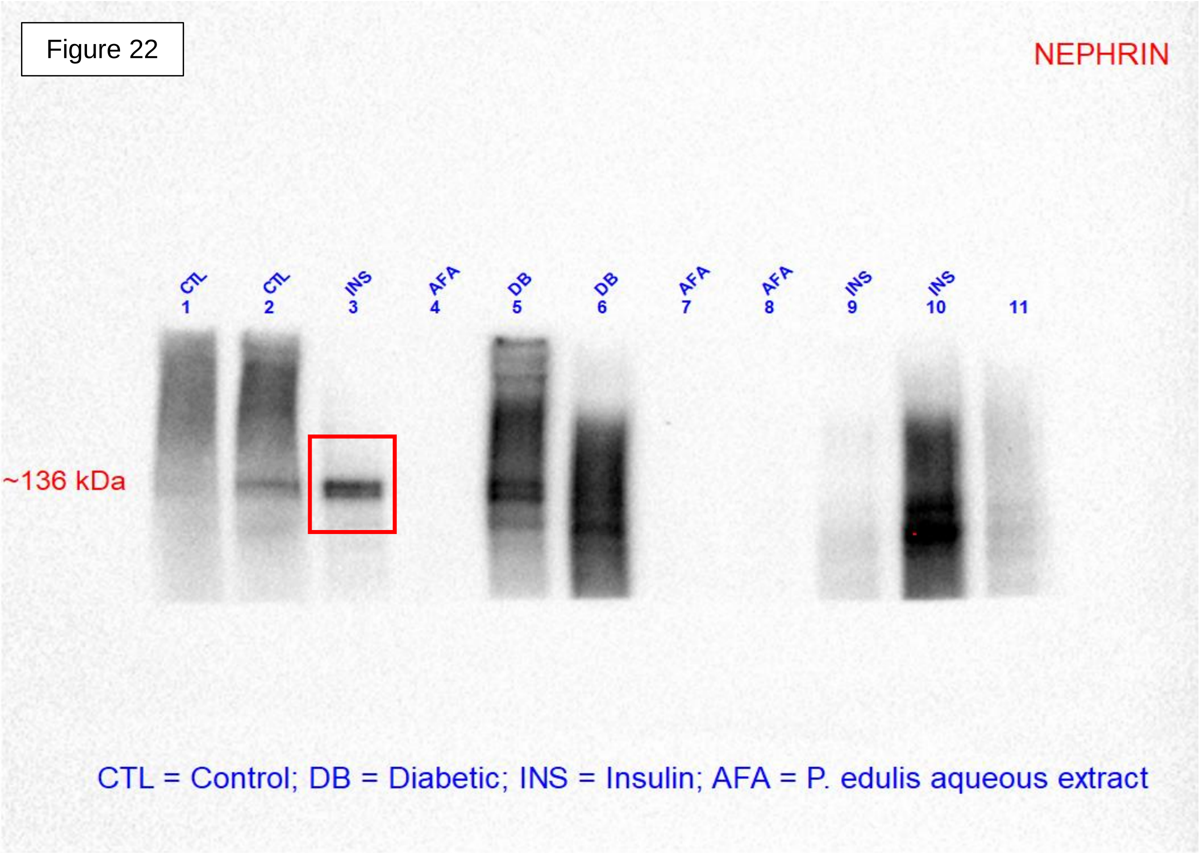

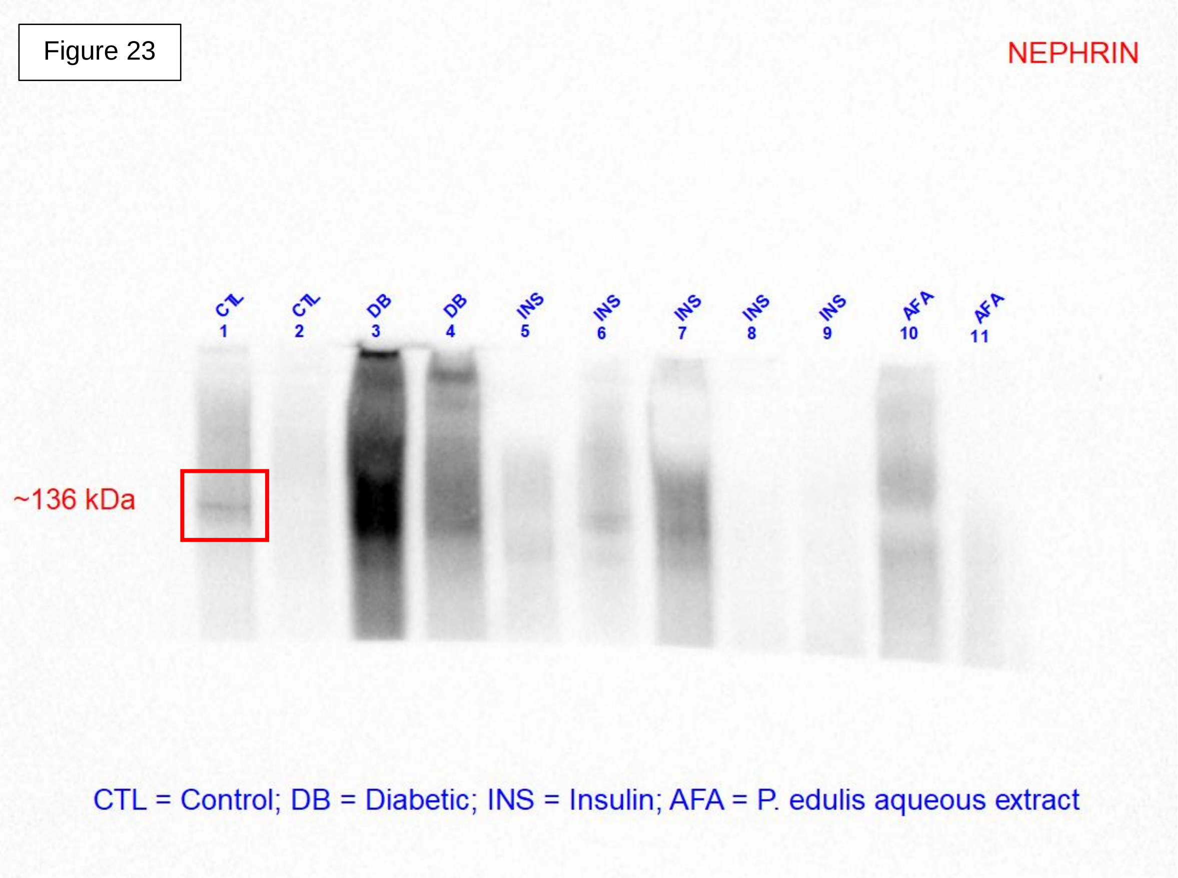

Figure 23

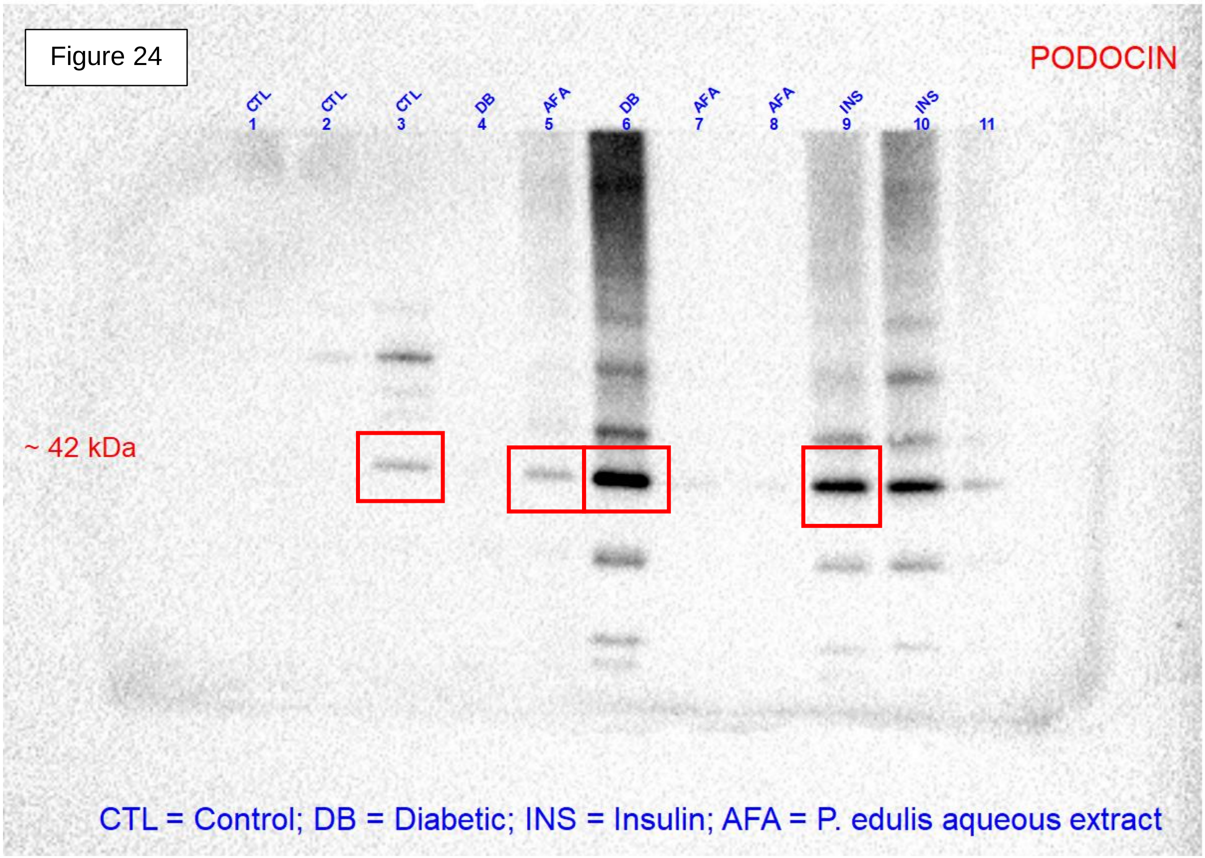

Figure 24

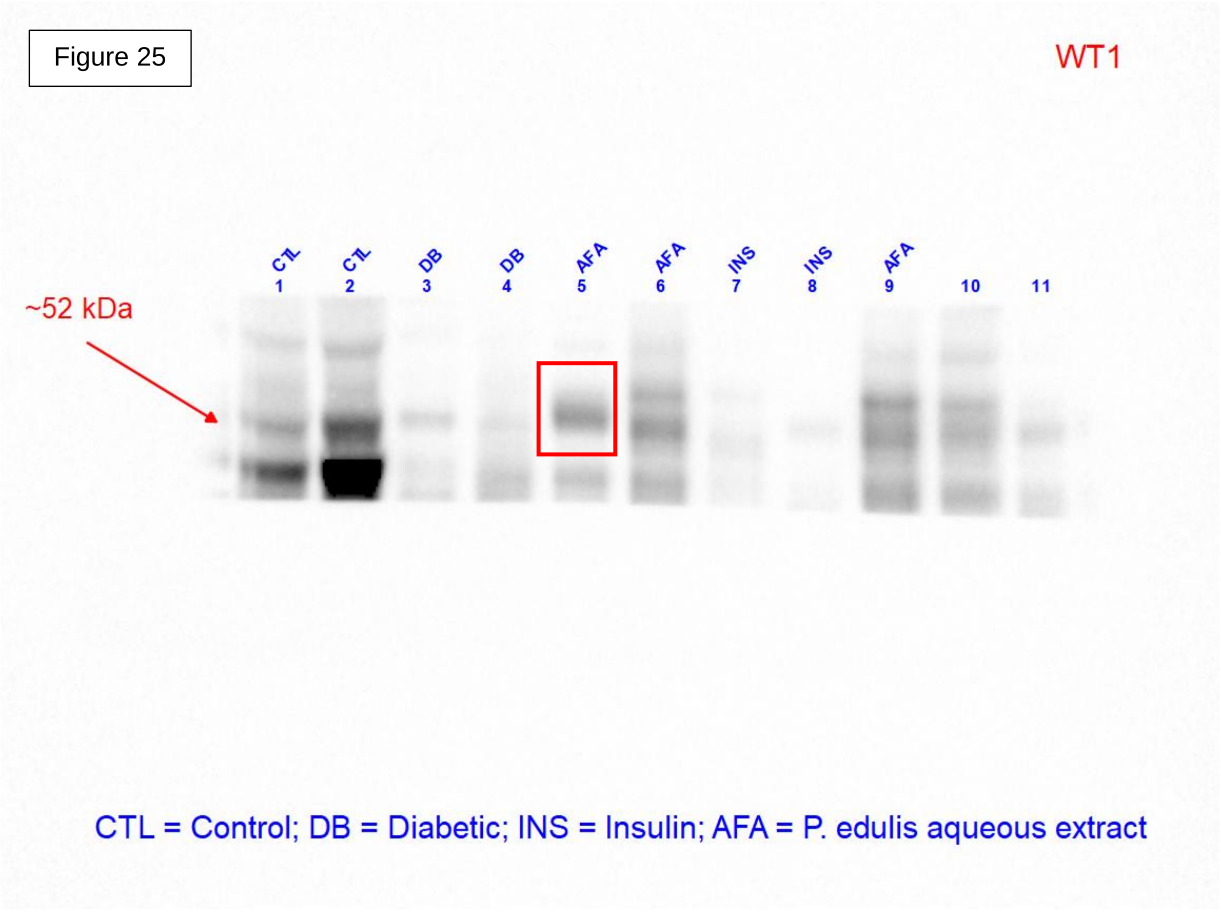

Figure 25

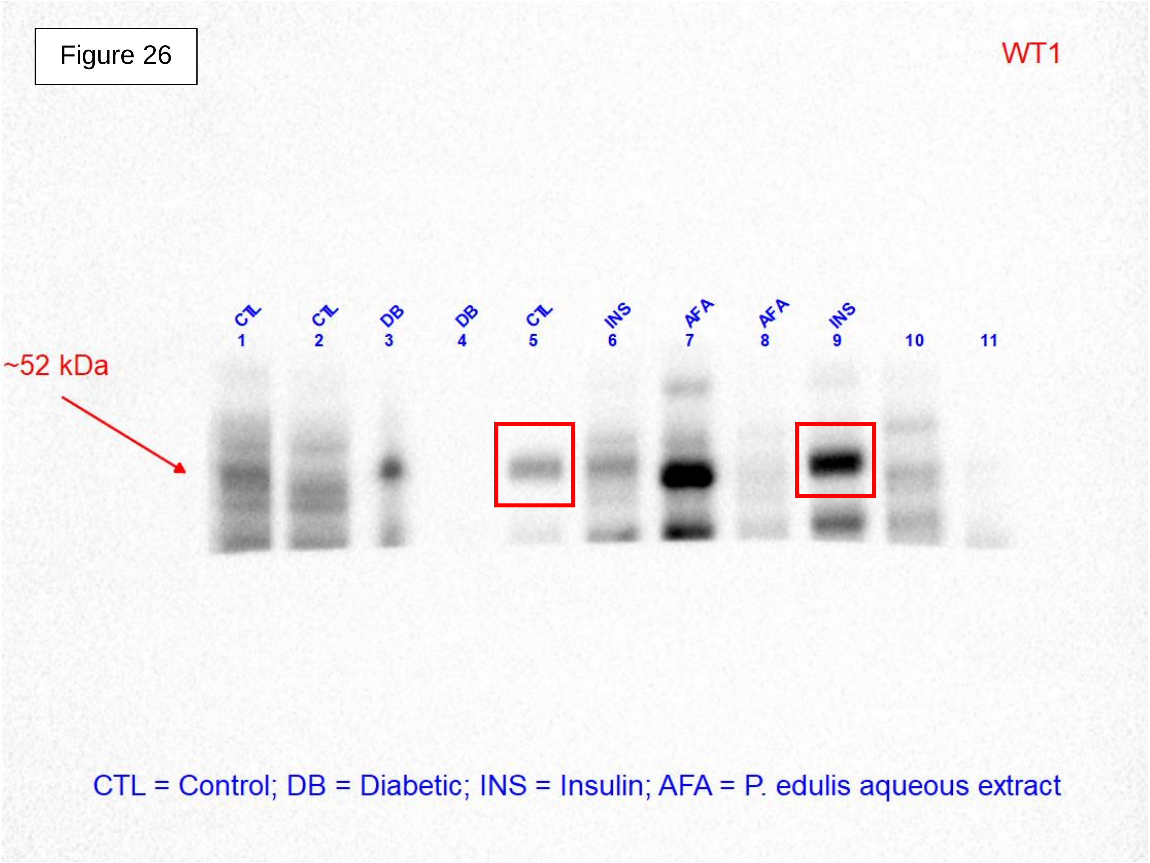

Figure 26

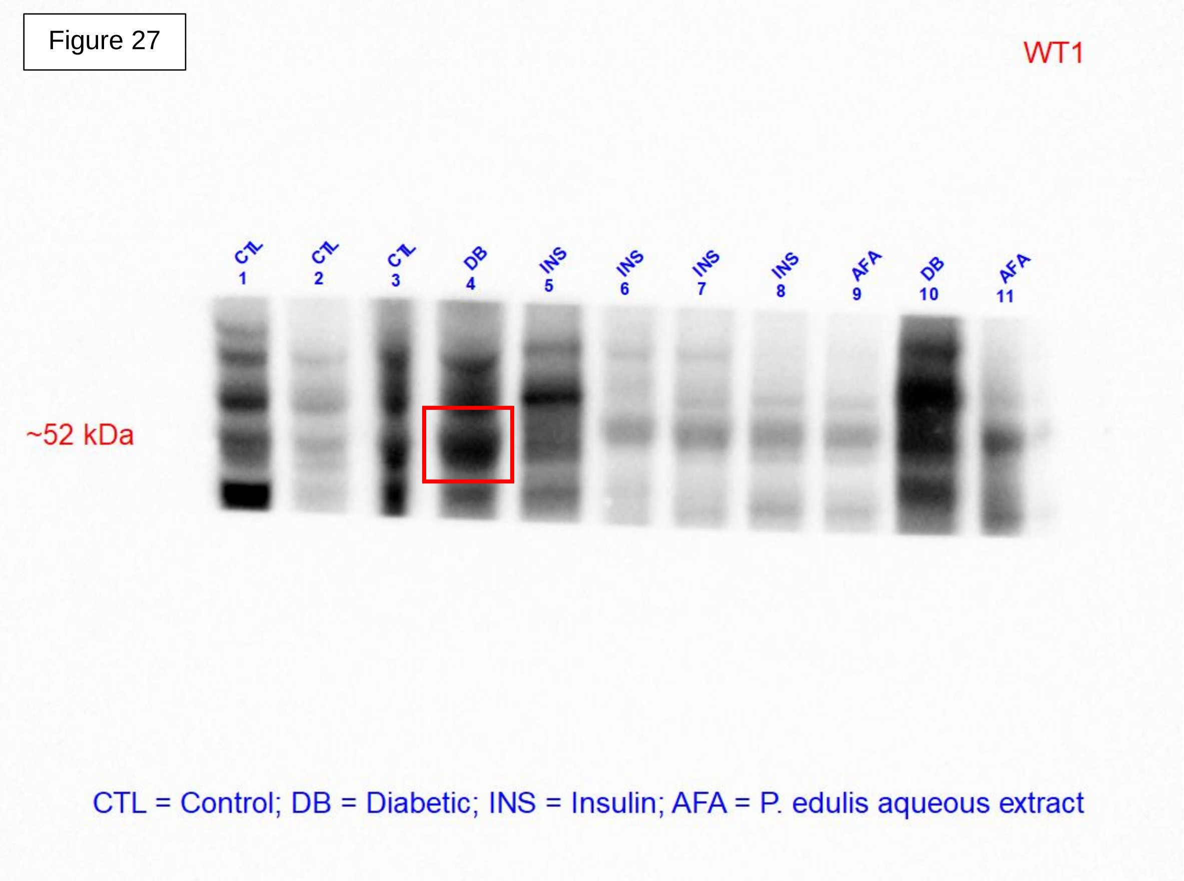
Figure 27
WT1
CTL 1
CTL 2
CTL 3
DB 4
INS 5
INS 6
INS 7
INS 8
AFA 9
DB 10
AFA 11
~52 kDa
CTL = Control; DB = Diabetic; INS = Insulin; AFA = P. edulis aqueous extract

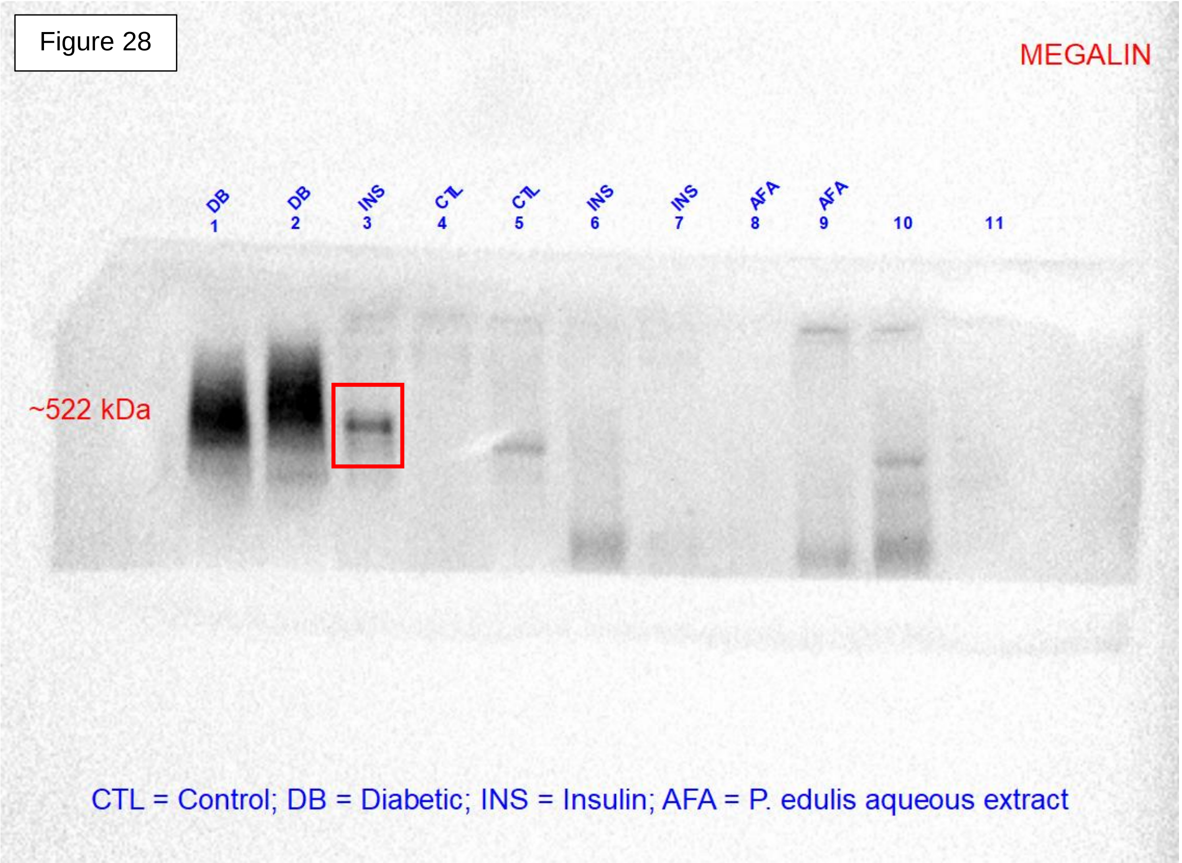
Figure 28
MEGALIN
DB 1
DB 2
INS 3
CTL 4
CTL 5
INS 6
INS 7
AFA 8
AFA 9
10
11
~522 kDa
CTL = Control; DB = Diabetic; INS = Insulin; AFA = P. edulis aqueous extract

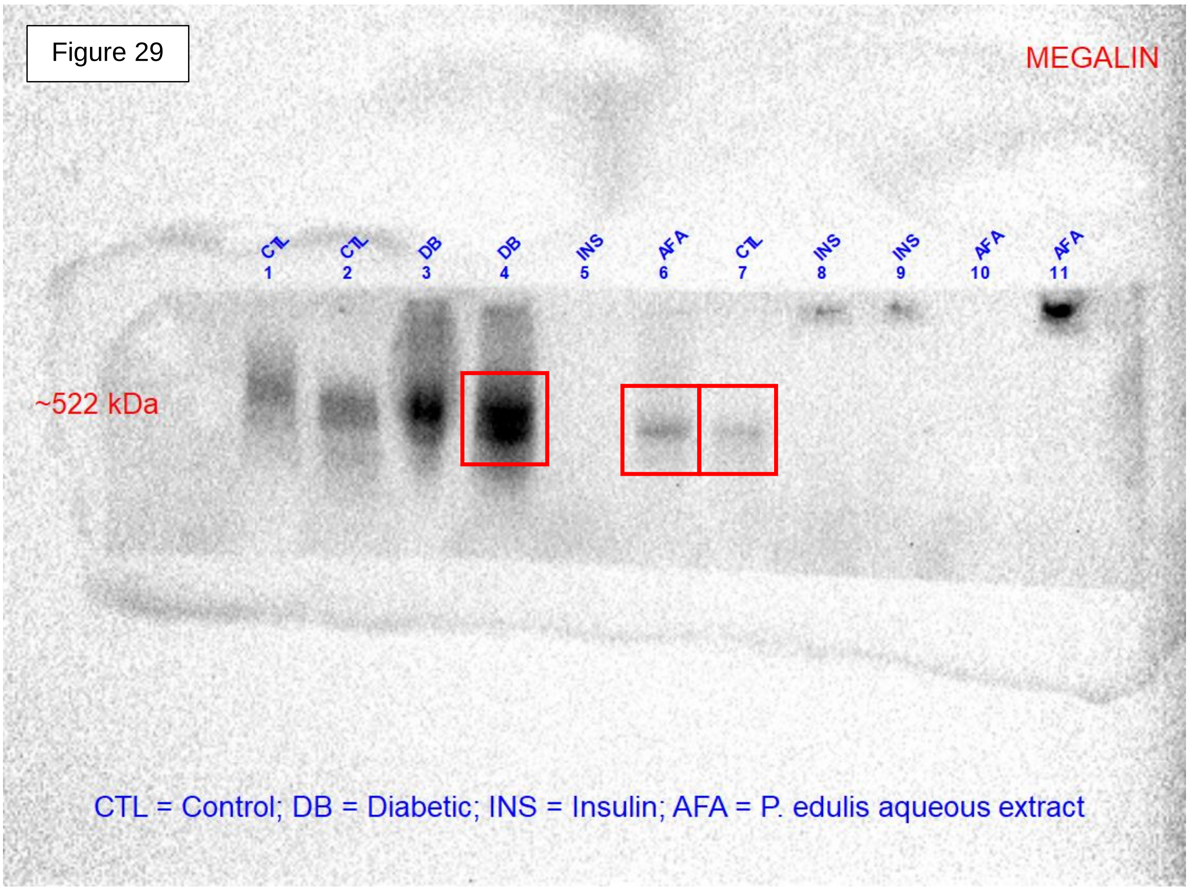
Figure 29
MEGALIN
CTL 1
CTL 2
DB 3
DB 4
INS 5
AFA 6
CTL 7
INS 8
INS 9
AFA 10
AFA 11
~522 kDa
CTL = Control; DB = Diabetic; INS = Insulin; AFA = P. edulis aqueous extract

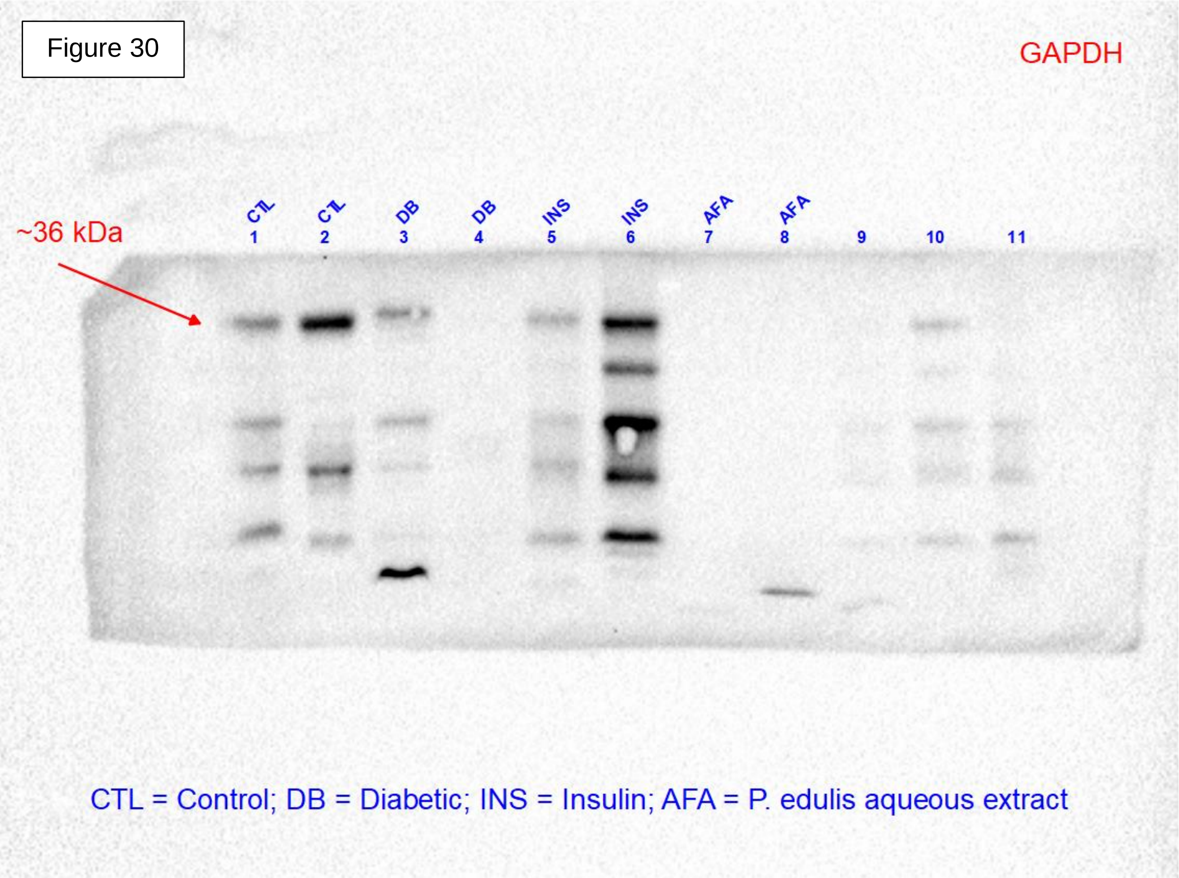
Figure 30
GAPDH
~36 kDa
CTL 1
CTL 2
DB 3
DB 4
INS 5
INS 6
AFA 7
AFA 8
9
10
11
CTL = Control; DB = Diabetic; INS = Insulin; AFA = P. edulis aqueous extract

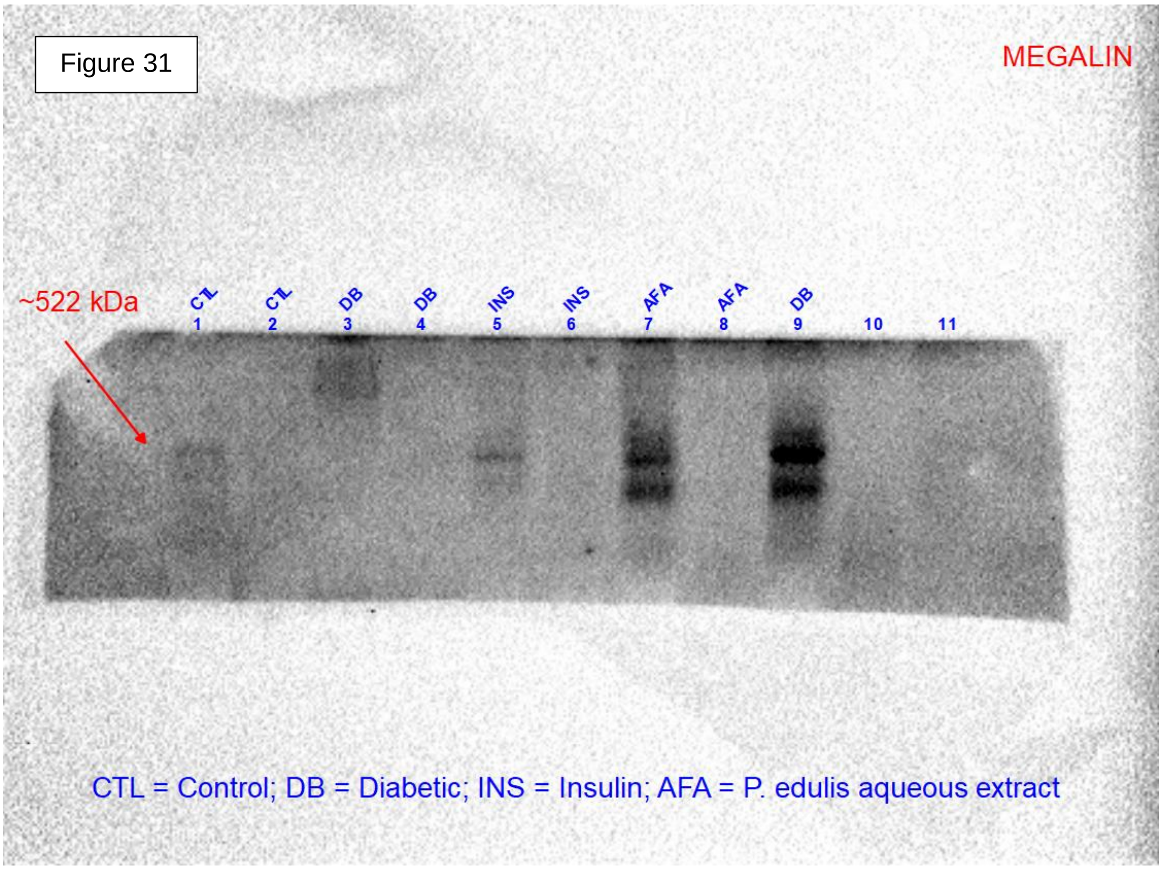
Figure 31
MEGALIN
~522 kDa
CTL 1
CTL 2
DB 3
DB 4
INS 5
INS 6
AFA 7
AFA 8
DB 9
10
11
CTL = Control; DB = Diabetic; INS = Insulin; AFA = P. edulis aqueous extract

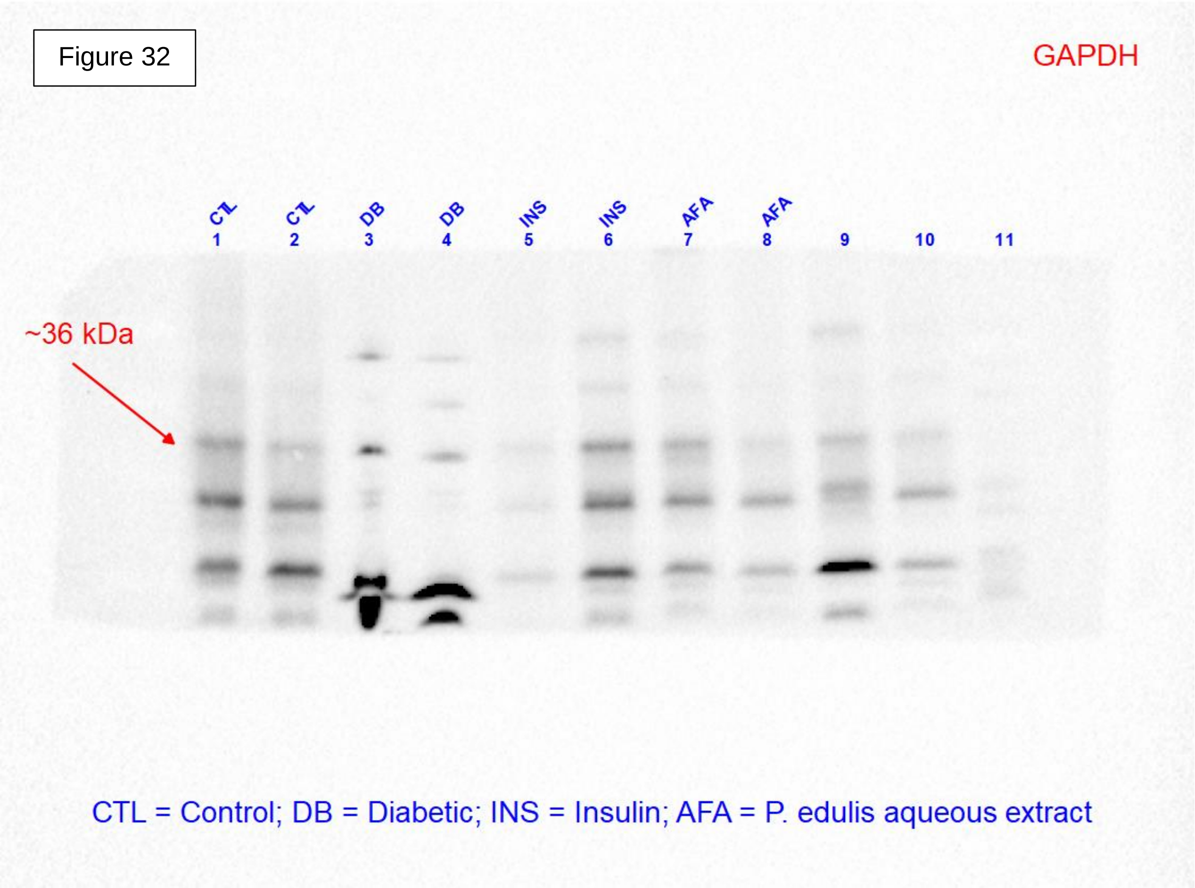
Figure 32
GAPDH
~36 kDa
CTL 1
CTL 2
DB 3
DB 4
INS 5
INS 6
AFA 7
AFA 8
9
10
11
CTL = Control; DB = Diabetic; INS = Insulin; AFA = P. edulis aqueous extract

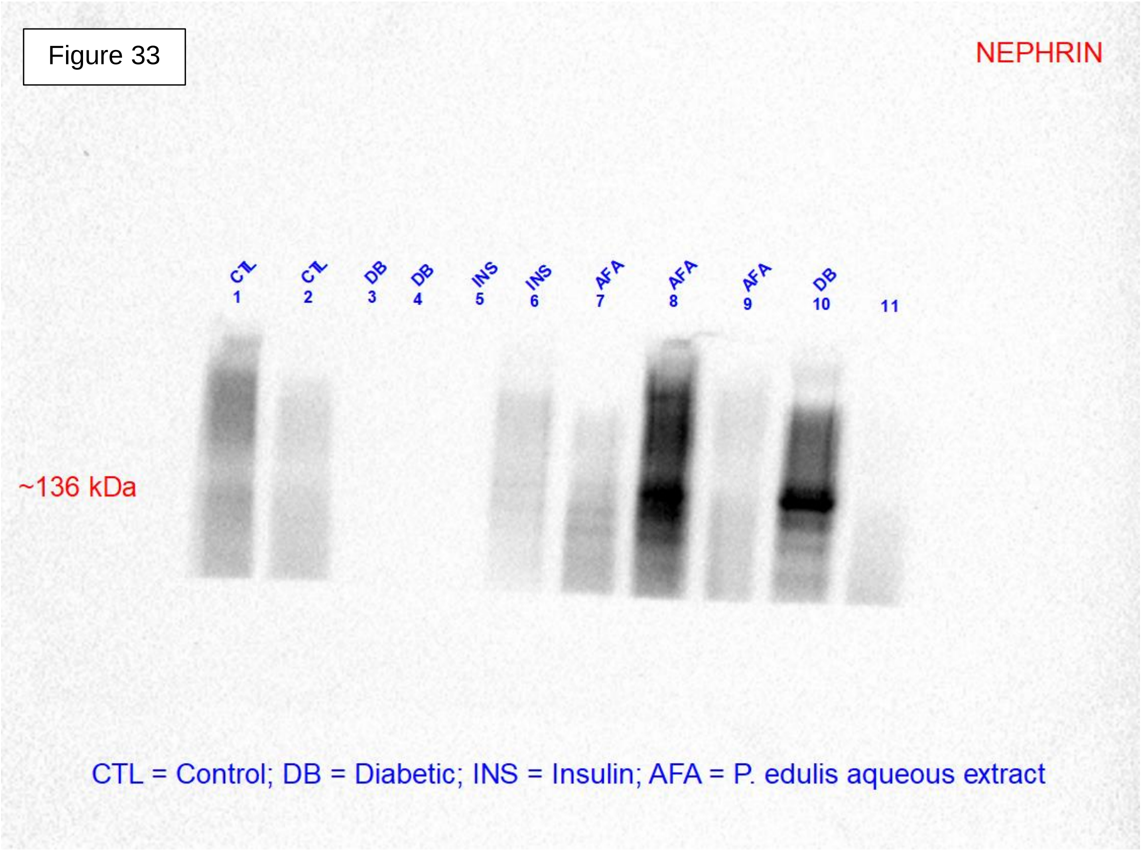
Figure 33
NEPHRIN
CTL 1
CTL 2
DB 3
DB 4
INS 5
INS 6
AFA 7
AFA 8
AFA 9
DB 10
11
~136 kDa
CTL = Control; DB = Diabetic; INS = Insulin; AFA = P. edulis aqueous extract

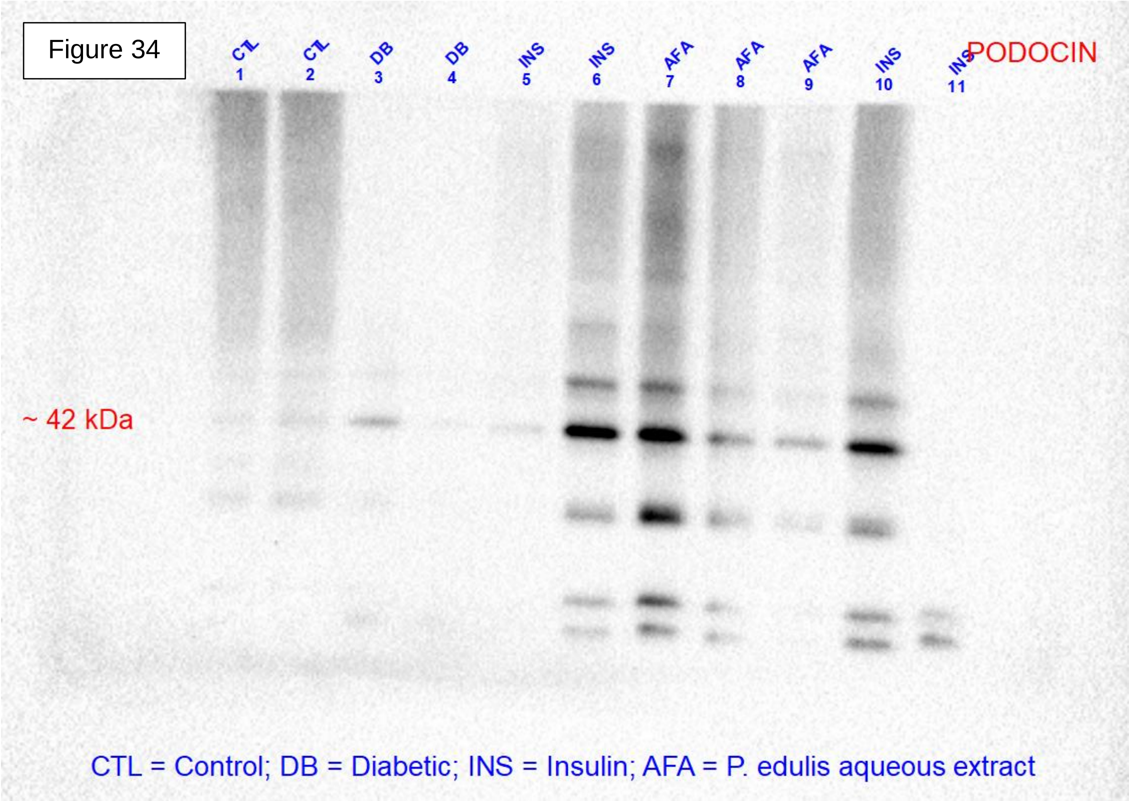
Figure 34
PODOCIN
CTL 1
CTL 2
DB 3
DB 4
INS 5
INS 6
AFA 7
AFA 8
AFA 9
INS 10
INS 11
~ 42 kDa
CTL = Control; DB = Diabetic; INS = Insulin; AFA = P. edulis aqueous extract

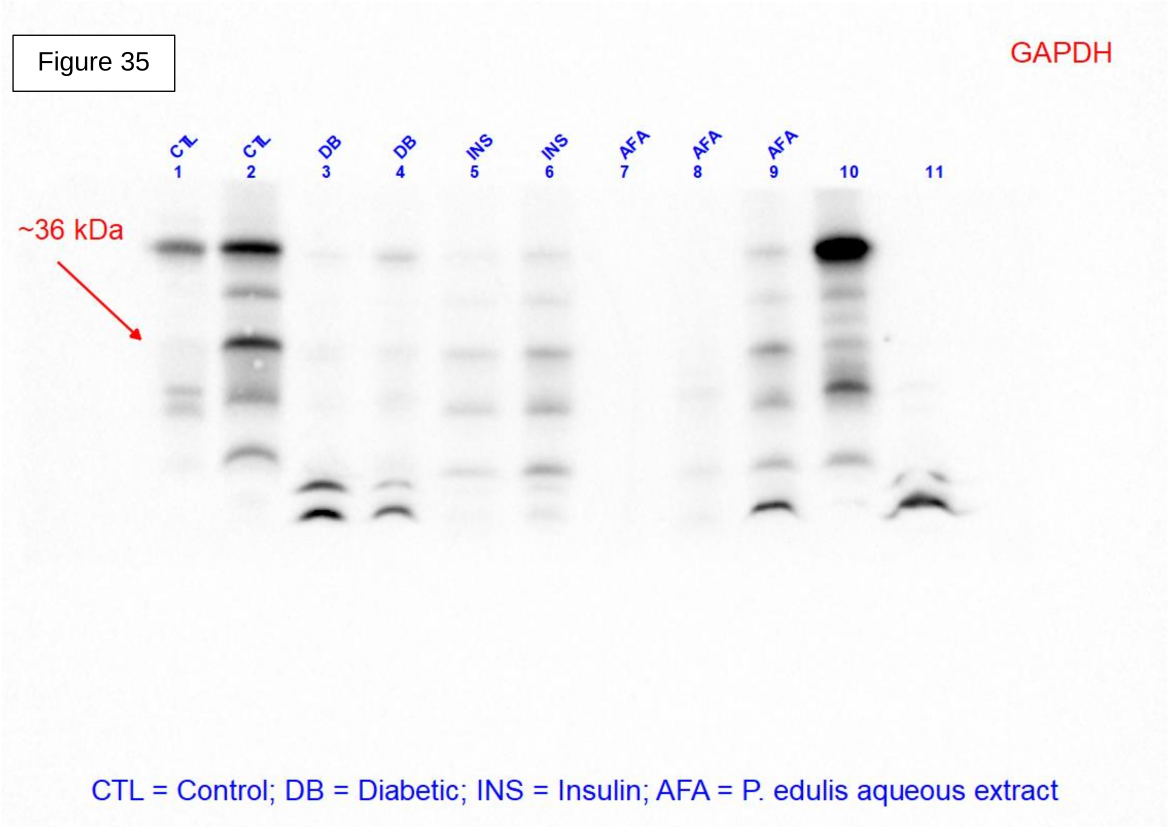
Figure 35
GAPDH
CTL 1
CTL 2
DB 3
DB 4
INS 5
INS 6
AFA 7
AFA 8
AFA 9
10
11
~36 kDa
CTL = Control; DB = Diabetic; INS = Insulin; AFA = P. edulis aqueous extract

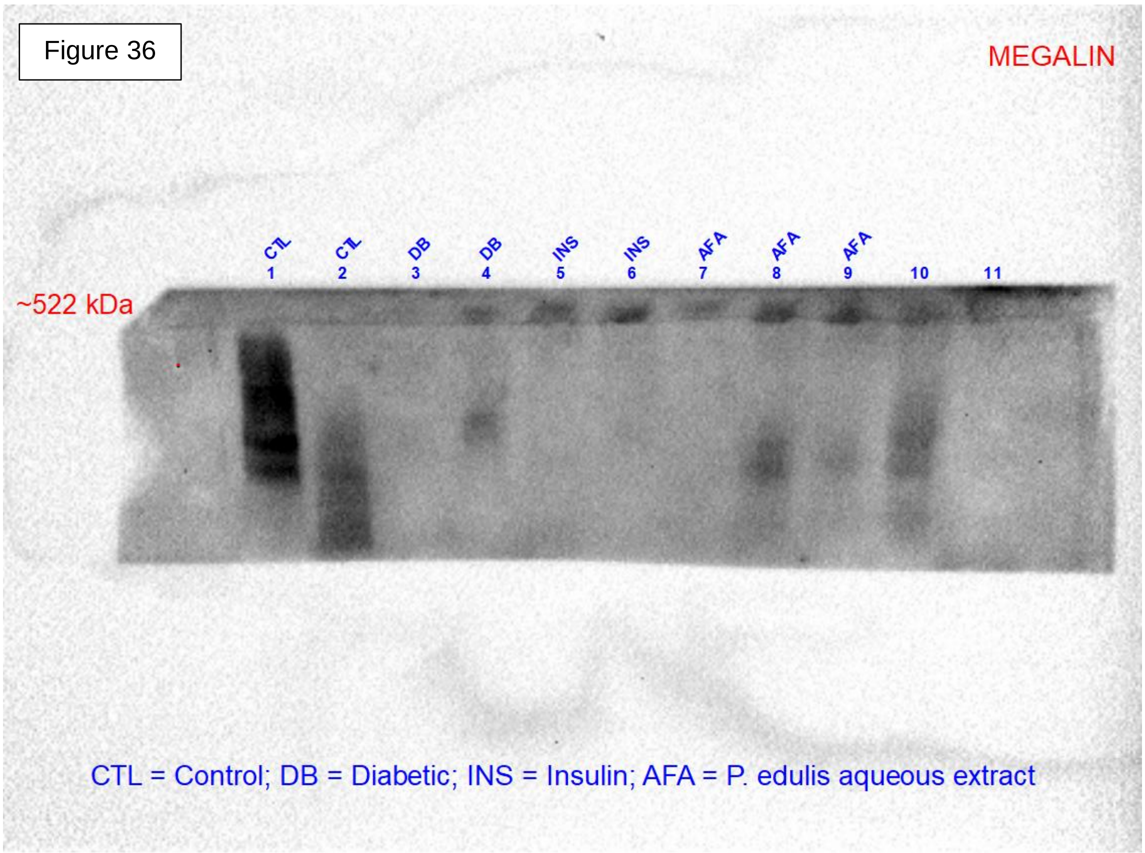
Figure 36
MEGALIN
CTL 1
CTL 2
DB 3
DB 4
INS 5
INS 6
AFA 7
AFA 8
AFA 9
10
11
~522 kDa
CTL = Control; DB = Diabetic; INS = Insulin; AFA = P. edulis aqueous extract

Figure 37

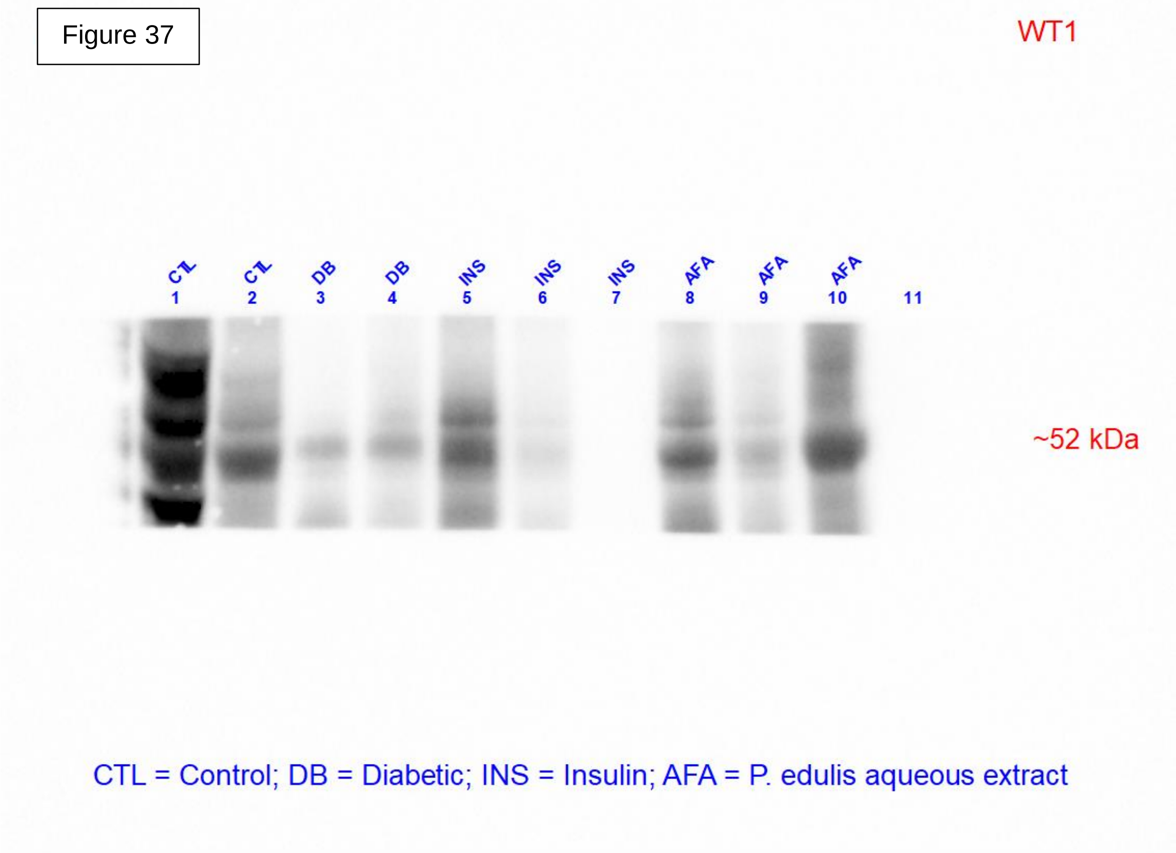

Figure 38

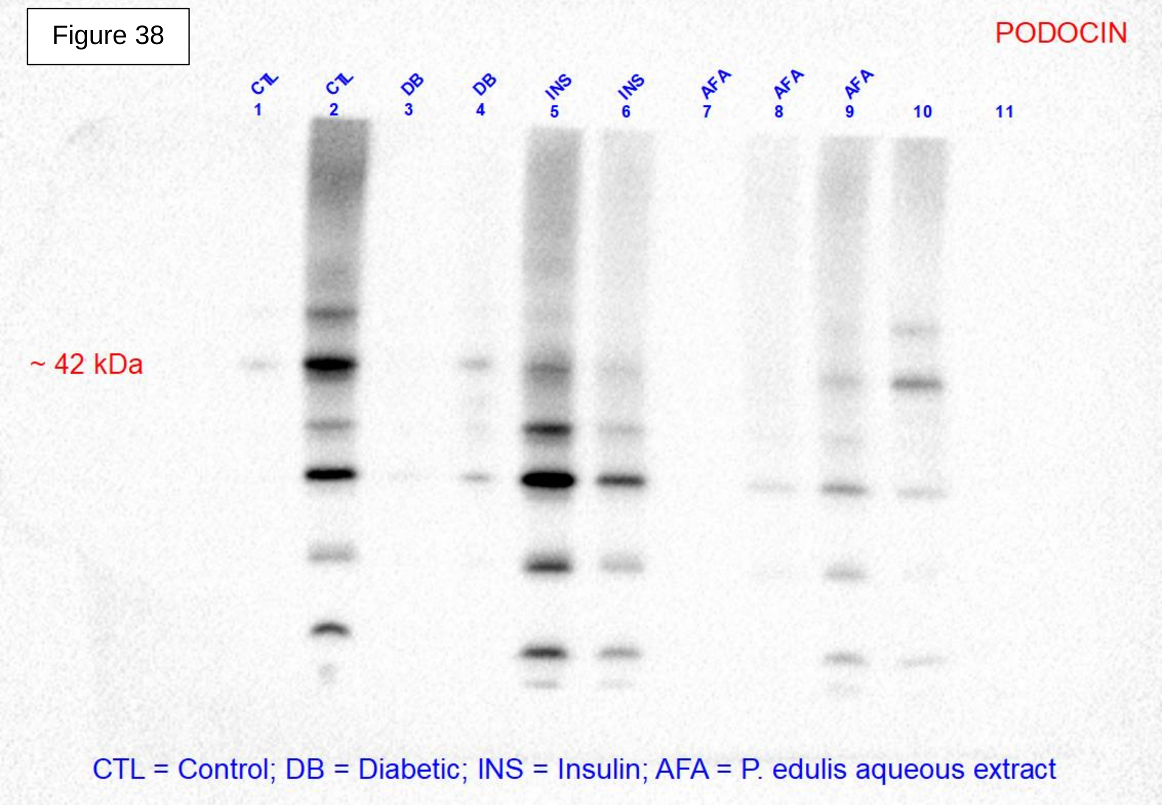

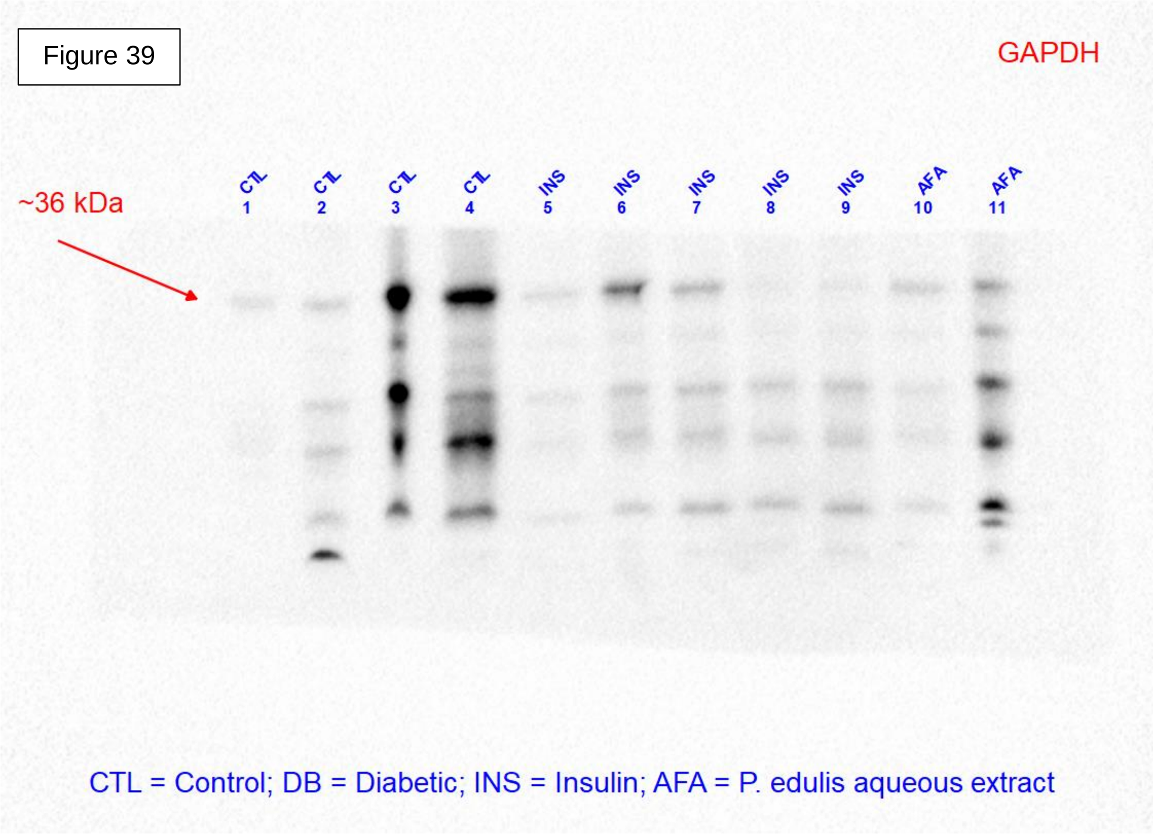
Figure 39
GAPDH
~36 kDa
CTL 1
CTL 2
CTL 3
CTL 4
INS 5
INS 6
INS 7
INS 8
INS 9
AFA 10
AFA 11
CTL = Control; DB = Diabetic; INS = Insulin; AFA = P. edulis aqueous extract

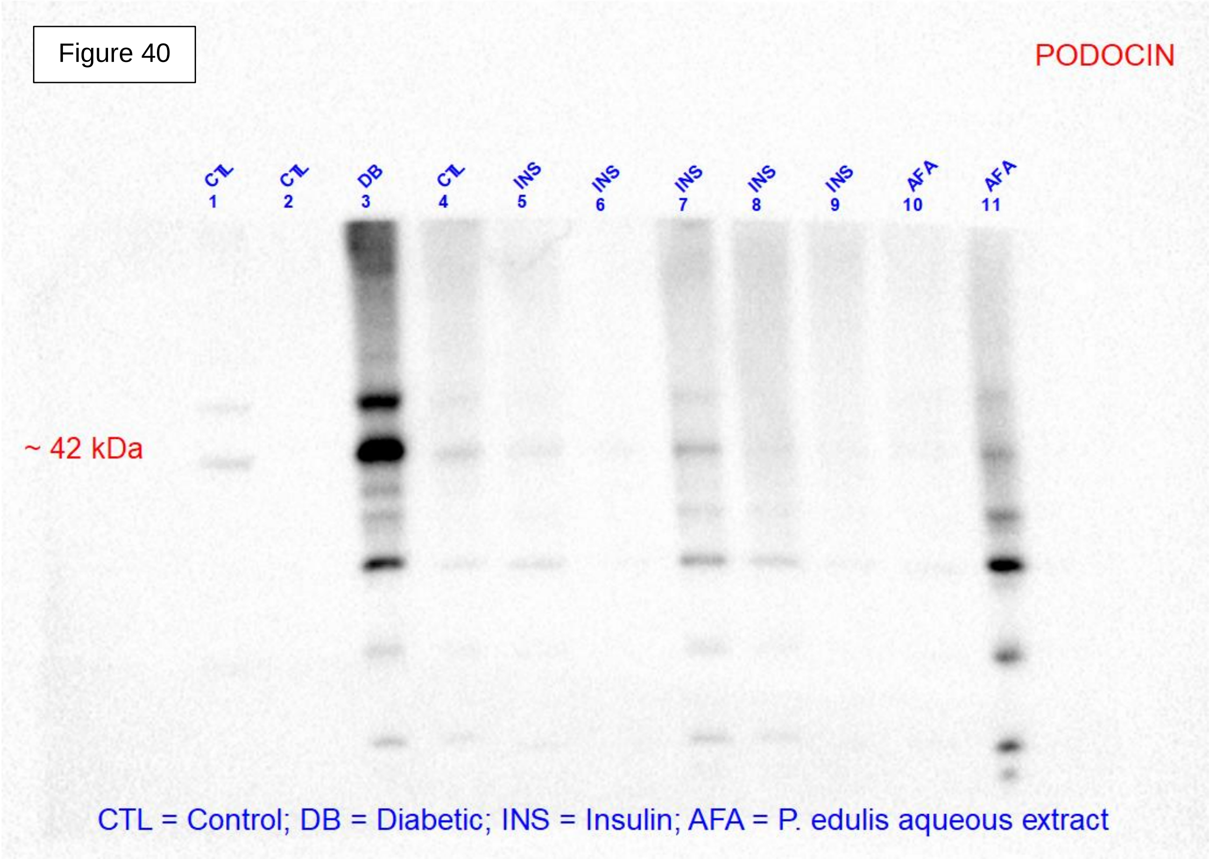
Figure 40
PODOCIN
~ 42 kDa
CTL 1
CTL 2
DB 3
CTL 4
INS 5
INS 6
INS 7
INS 8
INS 9
AFA 10
AFA 11
CTL = Control; DB = Diabetic; INS = Insulin; AFA = P. edulis aqueous extract
